# A Large Genome-Wide Association Study of Age-Related Hearing Impairment Using Electronic Health Records

**DOI:** 10.1371/journal.pgen.1006371

**Published:** 2016-10-20

**Authors:** Thomas J. Hoffmann, Bronya J. Keats, Noriko Yoshikawa, Catherine Schaefer, Neil Risch, Lawrence R. Lustig

**Affiliations:** 1 Department of Epidemiology and Biostatistics, and Institute for Human Genetics, University of California San Francisco, San Francisco, United States of America; 2 Department of Genetics, Louisiana State University Health Sciences Center, New Orleans, United States of America; 3 Department of Head and Neck Surgery, Kaiser Permanente Medical Center, Oakland, United States of America; 4 Kaiser Permanente Northern California Division of Research, Oakland, United States of America; 5 Department of Otolaryngology—Head and Neck Surgery, Columbia University Medical Center, Columbia, United States of America; 6 New York Presbyterian Hospital, New York, United States of America; National Institute on Deafness and Other Communication Disorders, National Institutes of Health, United States Of America

## Abstract

Age-related hearing impairment (ARHI), one of the most common sensory disorders, can be mitigated, but not cured or eliminated. To identify genetic influences underlying ARHI, we conducted a genome-wide association study of ARHI in 6,527 cases and 45,882 controls among the non-Hispanic whites from the Genetic Epidemiology Research on Adult Health and Aging (GERA) cohort. We identified two novel genome-wide significant SNPs: rs4932196 (odds ratio = 1.185, p = 4.0x10^-11^), 52Kb 3’ of *ISG20*, which replicated in a meta-analysis of the other GERA race/ethnicity groups (1,025 cases, 12,388 controls, p = 0.00094) and in a UK Biobank case-control analysis (30,802 self-reported cases, 78,586 controls, p = 0.015); and rs58389158 (odds ratio = 1.132, p = 1.8x10^-9^), which replicated in the UK Biobank (p = 0.00021). The latter SNP lies just outside exon 8 and is highly correlated (r^2^ = 0.96) with the missense SNP rs5756795 in exon 7 of *TRIOBP*, a gene previously associated with prelingual nonsyndromic hearing loss. We further tested these SNPs in phenotypes from audiologist notes available on a subset of GERA (4,903 individuals), stratified by case/control status, to construct an independent replication test, and found a significant effect of rs58389158 on speech reception threshold (SRT; overall GERA meta-analysis p = 1.9x10^-6^). We also tested variants within exons of 132 other previously-identified hearing loss genes, and identified two common additional significant SNPs: rs2877561 (synonymous change in *ILDR1*, p = 6.2x10^-5^), which replicated in the UK Biobank (p = 0.00057), and had a significant GERA SRT (p = 0.00019) and speech discrimination score (SDS; p = 0.0019); and rs9493627 (missense change in *EYA4*, p = 0.00011) which replicated in the UK Biobank (p = 0.0095), other GERA groups (p = 0.0080), and had a consistent significant result for SRT (p = 0.041) and suggestive result for SDS (p = 0.081). Large cohorts with GWAS data and electronic health records may be a useful method to characterize the genetic architecture of ARHI.

## Author Summary

Age-related hearing impairment (ARHI) is one of the most common sensory disorders. While ARHI effects can be mitigated with current technologies, it cannot be cured or eliminated. It is thus hoped that identification of genetic influences on ARHI may one day lead to curative therapies. Towards this goal, the current study utilized electronic health record data from non-Hispanic whites in the Genetic Epidemiology Research on Adult Health and Aging (GERA) cohort to conduct a genome-wide association study of ARHI, and tested the significant variants for replication in other GERA race/ethnicity groups, independent GERA phenotypes, and self-reported ARHI from the UK Biobank. We discovered two genome-wide significant SNPs. The first was novel and near *ISG20*. The second was in *TRIOBP*, a gene previously associated with prelingual nonsyndromic hearing loss. Motivated by our *TRIOBP* results, we also looked at exons in known hearing loss genes, and identified two additional SNPs, rs2877561 in *ILDR1* and rs9493672 in *EYA4* (at a significance threshold adjusted for number of SNPs in those regions). These results suggest that large cohorts with GWAS data and electronic health records may be a useful method to characterize the genetic architecture of ARHI.

## Introduction

Age-related hearing impairment (ARHI), or presbycusis, is one of the most common sensory impairments [1,2], affecting 25% of individuals over age 65 and 50% of individuals over age 80 [3]. ARHI impacts speech understanding, makes it much more difficult to communicate, and leads to an overall lower quality of life [4]. The effects of ARHI can be mitigated by amplification devices and assistive listening devices, but it is not curable and the effects cannot be completely eliminated. The best hope for cure lies in identifying all the physiologic and environmental factors contributing to ARHI and developing interventions that address these risks. There are a number of contributing factors to ARHI, including early noise exposure, medication history, and genetics [1,5–11]. It is hoped that by identifying the genetic basis of ARHI, targeted therapies can be developed to mitigate this risk.

ARHI has a clear genetic contribution; in a study of twins, hearing loss after age 64 was shown to have a heritability of 47% [12]; another study based on self-reported hearing loss in twins over age 75 reported a heritability of 40% [13]. During the past decade, genome-wide association studies (GWAS) have tried to uncover risk variants for ARHI, but no genome-wide significant or suggestive loci have been found that have been successfully replicated [3,14–16]. One study was based on 846 ARHI cases and 846 controls [3], while three other studies used principal components (PCs) of hearing impairment thresholds at several frequencies in 3,417 European individuals [15], 2,161 Belgian individuals [14], and 352 Finnish Saami individuals [16]. Fransen et al. attempted to replicate suggestive SNPs from previous papers, but failed to do so [14]. Of note, the sample sizes in these studies are smaller than those used to study other complex traits [17].

It is clear from these prior studies that much larger sample sizes are needed to better delineate the genetic factors associated with ARHI. Towards this goal, we utilized 6,527 non-Hispanic white ARHI cases and 45,882 controls identified from electronic health records (EHR) of members of the Kaiser Permanente Northern California (KPNC) health care system who participated in the Kaiser Permanente Research Program on Genes, Environment, and Health (RPGEH) Genetic Epidemiology Research on Adult Health and Aging (GERA) cohort for discovery. Loci with genome-wide significance identified in the GERA non-Hispanic whites were then tested for independent replication in 1,025 Latino, East Asian, and African American GERA cases and 12,388 controls, in addition to 30,802 UK Biobank self-reported cases and 78,586 controls. We also tested for replication in two related measured quantitative traits—speech discrimination score (SDS) and speech recognition threshold (SRT), stratified by case/control status, to construct independent tests. These measures were only available on a subset of the GERA individuals. Additionally, we assessed replication of previously-described sub-genome-wide significant loci in the GERA cohort. Motivated by our GWAS results, we also examined the GWAS results at known hearing loss genes at reduced significance thresholds adjusted for the proportion of the genome being tested to account for lack of power in these regions. Finally, we looked at array-based heritability in the GERA non-Hispanic whites.

## Results

### The GERA cohort

The multi-ethnic GERA cohort participants in this study were part of the Kaiser Permanente Research Program on Genes, Environment, and Health (RPGEH), which has been described in detail [18,19]. Briefly, participants were an average of 62.9 years old (SD = 13.8) at sample collection, with an average length of membership of 23.5 years in Kaiser Permanente Northern California, comprehensive EHR, survey (five pages including information on demographic factors, behaviors, and self-reported health, but no questions on hearing), and genome-wide genotyping data.

**Fig 1:**
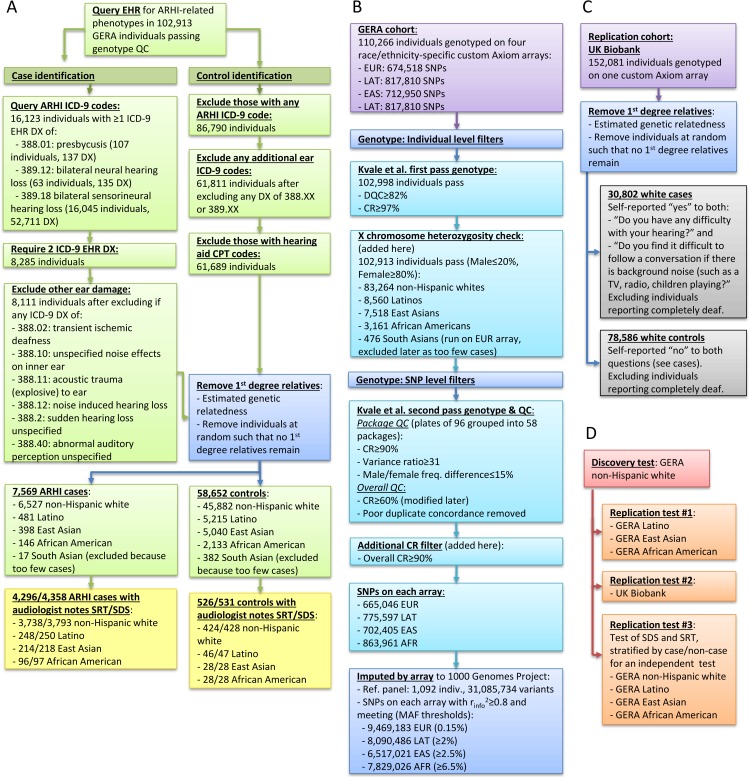
Study design. (A) GERA genotyping, (B) GERA phenotyping, (C) UK Biobank replication cohort, and (D) analysis. DX, diagnosis.

We used as our discovery cohort the largest GERA subgroup of non-Hispanic whites (6,527 cases, 45,882 controls), with our replication cohort in GERA consisting of Latinos/East Asians/African Americans with 481/398/146 cases and 5,215/5,040/2,133 controls, respectively (workflow of GERA phenotyping in Fig 1A). Compared to the controls, the cases were more often male and older (Table 1). There were 4,903 total individuals (4,231 non-Hispanic white, 298 Latino, 249 East Asian, and 125 African American) for whom audiologist notes were available (the majority in cases), and could be used to derive quantitative measures of speech recognition threshold (SRT) and speech discrimination score (SDS), as well as history of noise exposure (Table 1).

**Table 1:** Characteristics of age-related hearing impairment (ARHI) cases and controls in the GERA cohort. Note that speech reception threshold (SRT) and speech discrimination score (SDS) were only available on a small portion of the cohort. For dichotomous variables, N (% of non-missing), and for continuous variables, mean (sd), unless otherwise indicated.

	Non-Hispanic white		Latino		East Asian		African American	
	Case	Control	Case	Control	Case	Control	Case	Control
ARHI	6527 (12.5%)	45882	481 (8.4%)	5215	398 (7.3%)	5040	146 (6.4%)	2133
Female	3087 (47.3%)	28454 (62.0%)	212 (44.1%)	3312 (63.5%)	156 (39.2%)	3047 (60.5%)	73 (50.0%)	1277 (59.9%)
Age	75.1 (9.1)	67.0 (13.0)	73.2 (9.5)	60.5 (14.1)	74.6 (9.5)	61.1 (13.9)	75.1 (9.7)	64.9 (13.2)
SRT N	3738 (57.3%)	424 (0.9%)	248 (51.6%)	46 (0.9%)	214 (53.8%)	28 (0.6%)	96 (65.8%)	28 (1.3%)
SRT # Meas	2.0 (1.2)	1.1 (0.2)	2.1 (1.5)	1.1 (0.3)	2.0 (1.1)	1.1 (0.3)	1.8 (1.0)	1.2 (0.8)
SRT Median [IQR]	32.5 [22.5–43.8]	10.0 [5.0–15.0]	32.5 [25.0–42.5]	7.5 [5.0–12.5]	35.0 [25.0–47.5]	10.0 [7.2–13.1]	35.0 [25.0–42.5]	10.6 [10.0–15.0]
SDS N	3793 (58.1%)	428 (0.9%)	250 (52.0%)	47 (0.9%)	218 (54.8%)	28 (0.6%)	97 (66.4%)	28 (1.3%)
SDS # Meas	2.0 (1.2)	1.1 (0.2)	2.1 (1.5)	1.1 (0.3)	2.0 (1.2)	1.1 (0.3)	1.8 (1.1)	1.2 (0.8)
SDS Median [IQR]	90.0 [79.0–96.0]	100.0 [100.0–100.0]	88.0 [74.0–96.0]	100.0 [100.0–100.0]	86.0 [73.4–96.0]	100.0 [100.0–100.0]	92.0 [82.0–96.0]	100.0 [100.0–100.0]
Noise N	3826 (58.6%)	429 (0.9%)	254 (52.8%)	47 (0.9%)	220 (55.3%)	29 (0.6%)	97 (66.4%)	28 (1.3%)
Noise	2213 (57.8%)	194 (45.2%)	153 (60.2%)	21 (44.7%)	129 (58.6%)	11 (37.9%)	54 (55.7%)	10 (35.7%)

Within each race/ethnicity group, we also tested ancestry principal components (PCs) [18] for association with ARHI, adjusting for the potential confounders of diabetes, hypertension, and osteoporosis. In non-Hispanic whites, we found a significant increase in ARHI in those with northwestern vs. southeastern European ancestry (p = 9.2×10^−10^), but it explained very little of the variance (0.12%). No significant associations were found in Latinos, East Asians, or African Americans, but we had less statistical power in these groups.

### GWAS results

**Fig 2:**
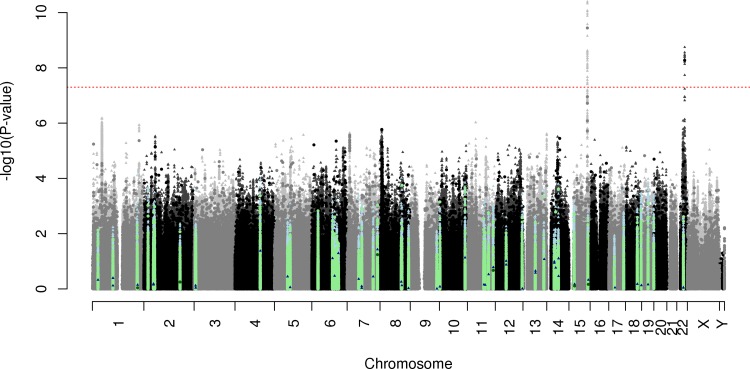
ARHI Manhattan plot in GERA non-Hispanic whites. SNPs meeting genome-wide significance (p<5x10^-8^) are above the red line. The circles indicate genotyped SNPs, and the triangles indicate imputed SNPs. Dark colored points indicate previously-described sub-threshold suggestive hits, and light colored points are within 0.5Mb of the actual SNP (blue imputed, green genotyped).

In the discovery stage of the GWAS, we analyzed the GERA non-Hispanic whites (workflow of genotyping of GERA and replication cohorts in Fig 1B and 1C and of analysis in Fig 1D, Manhattan plot in Fig 2, Q-Q plot in S1 Fig). The genomic inflation factor was reasonable for the sample size being tested (genotyped λ = 1.037, imputed λ = 1.053) [20]. We detected two novel genome-wide significant (p<5x10^-8^) loci associated with ARHI.

**Fig 3:**
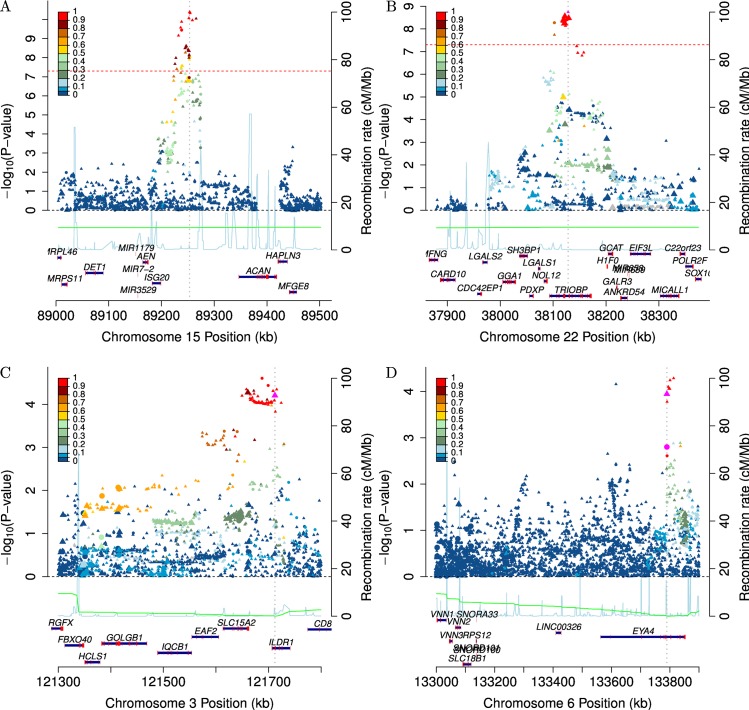
Zoomed in locus plots in GERA non-Hispanic whites. The circles indicate genotyped SNPs, and the triangles indicate imputed SNPs. The blue line is the recombination map, which is given according to the y-axis on the right hand side of the plot. The color scheme identifies the correlation to the lead SNP in the locus (colorbar on the left). Larger points indicate SNPs in exons. Genome-wide significant SNPs are given in (A) rs4932196 and (B) rs58389158, and SNPs identified in hearing loss gene exons in (C) rs2877561 and (D) rs9493627.

The first SNP, rs4932196, was at 15q26, b37 position 89,253,268 (GERA non-Hispanic white odds ratio (OR) = 1.185, p = 4.0x10^-11^, frequency of the risk allele = 0.810, zoomed in plot of locus in Fig 3A), 52kb 3’ of *ISG20* and 638kb 5’ of *ACAN*. There were several genome-wide significant and suggestive typed SNPs around the top (imputed) SNP–rs6496519, p = 1.3x10^-10^, call rate (CR) = 99.9%, r^2^ = 0.953; rs35701059, p = 7.8x10^-9^, CR = 99.9%, r^2^ = 0.858; rs11073807, p = 1.5x10^-7^, CR = 99.8%, r^2^ = 0.389 –giving strong evidence that the signal was not driven by genotyping error. Further, the same top SNP was replicated in the meta-analysis of the GERA Latinos, East Asians, and African Americans (OR = 1.192, p = 0.00094, one-sided for all replication tests since the hypothesis is that the effect is in the same direction), and was in the same direction in each individual group (Table 2). The same SNP was also replicated in UK Biobank individuals (OR = 1.028, p = 0.015) although that analysis was based on a self-reported cross-sectional phenotype that was different from the GERA individuals (see Methods). Meta-analysis of all four GERA race/ethnicity groups together gave an OR = 1.186, p = 2.8x10^-13^, with no evidence of heterogeneity (I^2^ = 0, p = 0.82); we did not include the UK Biobank in the meta-analysis due to differences in the phenotype. On the subset of the cohort that had audiologist notes, the SNP association with overall transformed SRT was suggestive (β = 0.043, higher values indicate worse hearing, p = 0.039, stratified by case/control status for an independent test, see Methods), and the association with overall SDS less so (β = 0.023, higher values indicate worse hearing, p = 0.13), although both were in the same direction as the ARHI phenotype (Table 2).

**Table 2:** Genome-wide significant GWAS SNPs. The genotype is coded for the risk increasing allele, which is the first one mentioned (e.g., T in T/A). The discovery test of ARHI in GERA non-Hispanic whites was two-sided^#^, and all replication tests were one-sided. There are no estimates for African American cases SDS/SRT, as the sample size was too small. OR, odds ratio (for case/control phenotypes); Effect, effect size estimate (for SDS/SRT phenotypes); Freq, frequency of the risk increasing allele; SRT, speech reception threshold; SDS, speech discrimination score.

		rs4932196 (T/A)– 52kb 3’ of *ISG20*, 638kb 5’ *ACAN*				rs58389158 (T/TA)– 5773bp 3’ exon 7, 1021 bp 5’ exon 8 of long form of *TRIOBP*			
Phenotype	Group	Freq	OR/Effect	P	r_info_^2^	Freq	OR/Effect	P	r_info_^2^
ARHI ICD-9 case/control	GERA non-Hispanic white^#^	0.810	1.185	4.0x10^-11^	0.99	0.421	1.132	1.8x10^-9^	0.90
	GERA Latino	0.726	1.147	0.047	0.98	0.369	1.039	0.30	0.99
	GERA East Asian	0.525	1.215	0.0082	0.95	0.567	1.108	0.11	0.92
	GERA African American	0.930	1.504	0.079	0.98	0.221	0.850	0.86	0.97
	GERA Latino, East Asian, and African American replication meta-analysis	-	1.192	0.00094	-	-	1.041	0.22	-
ARHI case/control by self-report	UK Biobank	0.804	1.028	0.015	1.00	0.459	1.036	0.00021	1.00
Cases SRT, modeled as sqrt(SRT), higher is worse hearing	GERA non-Hispanic white	0.832	0.030	0.23	0.99	0.433	0.050	0.13	0.99
	GERA Latino	0.707	0.299	0.0079	0.98	0.345	0.248	0.019	0.98
	GERA East Asian	0.639	0.048	0.39	0.95	0.575	0.026	0.42	0.95
	GERA African American	-	-	-	0.98	-	-	-	0.98
	GERA Meta-analysis of all	-	0.056	0.070	-	-	0.059	0.029	-
Controls SRT	GERA non-Hispanic white	0.835	-0.006	0.57	0.99	0.431	0.125	1.8x10^-6^	0.99
	GERA Latino	0.779	0.227	0.013	0.98	0.369	-0.181	0.98	0.98
	GERA East Asian	0.578	0.145	0.078	0.95	0.550	0.218	0.015	0.95
	GERA African American	0.946	0.301	0.21	0.98	0.206	0.243	0.12	0.98
	GERA Meta-analysis of all	-	0.034	0.14	-	-	0.109	5.7x10^-6^	-
Overall SRT	GERA Meta-analysis of all	-	0.043	0.039	-	-	0.090	1.9x10^-6^	-
Cases SDS, modeled as log(100-SDS+1), higher is worse hearing	GERA non-Hispanic white	0.829	0.066	0.037	0.99	0.433	0.019	0.26	0.99
	GERA Latino	0.702	0.041	0.36	0.98	0.339	-0.098	0.85	0.98
	GERA East Asian	0.637	0.322	0.020	0.95	0.579	0.271	0.021	0.95
	GERA African American	-	-	-	0.98	-	-	-	0.98
	GERA Meta-analysis of all	-	0.076	0.013	-	-	0.019	0.24	-
Controls SDS	GERA non-Hispanic white	0.830	-0.030	0.84	0.99	0.430	0.013	0.28	0.99
	GERA Latino	0.778	0.122	0.081	0.98	0.371	-0.128	0.95	0.98
	GERA East Asian	0.560	0.100	0.15	0.95	0.557	-0.001	0.50	0.95
	GERA African American	0.947	-0.230	0.87	0.98	0.205	0.103	0.30	0.98
	GERA Meta-analysis of all	-	-0.009	0.63	-	-	0.002	0.46	-
Overall SDS	GERA Meta-analysis of all	-	0.023	0.13	-	-	0.009	0.30	-

The second SNP, rs58389158, is an imputed indel at 22q13.1, b37 position 38,128,283 (GERA non-Hispanic white OR = 1.132, p = 1.8x10^-9^, frequency of the risk allele = 0.421, zoomed in plot of locus in Fig 3B), in an intron of the long form of *TRIOBP* (Table 2). The signal initially appeared to be driven by one genotyped SNP, rs5750477 (p = 1.8x10^-8^), with a lower CR = 90.8%, and r^2^ = 0.77 with rs58389158. Although the CR was low, genotype cluster plots of the SNP showed well separated clusters with only modest overlap. However, to ensure the association was not driven by genotyping artifacts, we removed rs5750477 from the analysis and re-imputed rs58389158. The SNP did not impute quite as well as previously (r^2^ = 0.75 as opposed to r^2^ = 0.90 with the SNP), and had a slightly less significant p-value (p = 1.0x10^-7^), which likely reflects reduced power due to poorer imputation accuracy. Although the SNP did not show evidence of association in the replication meta-analysis of the GERA Latinos, East Asians, and African Americans (OR = 1.041, p = 0.22), the effect was in the same direction. In addition, the SNP association was replicated in the UK Biobank data (OR = 1.036, p = 0.00021), and was strongly associated with transformed SRT (β = 0.090, p = 1.9x10^-6^). For SDS, the SNP effect was in the same direction but not statistically significant (β = 0.009, p = 0.30).

We also looked for a sex difference in the OR of ARHI with the top SNPs in the GERA non-Hispanic whites. There was no significant evidence of heterogeneity (rs4932196 I^2^ = 55.4, p = 0.13; rs58389158 I^2^ = 66.9, p = 0.082) between males and females (rs4932196 OR_male_ = 1.143, OR_female_ = 1.234; rs58389159 OR_male_ = 1.166, OR_female_ = 1.085). Also, no additional loci were identified in sex-specific GWAS analyses. Finally, no additional SNPs at the two novel loci were discovered in a conditional analysis including the top two SNPs.

### Testing previously reported ARHI associations

We tested 58 previously reported sub-genome-wide significant SNPs [3,14–16] for replication in GERA non-Hispanic whites. No SNP reached a Bonferroni corrected threshold of 0.00086, with only three SNPs being of marginal significance 0.01<p<0.05 (S1 Table).

### ARHI associations at previously identified hearing loss genes

Motivated by our *TRIOBP* results, we also examined SNPs from our GWAS results in 132 known Mendelian hearing loss genes [21] in GERA non-Hispanic whites at a reduced significance threshold adjusted for the proportion of the genome being tested to account for potential lack of power in these regions. For each of the genes, we looked separately at exonic SNPs, specifically non-synonymous and synonymous coding changes, and then SNPs that were eQTLs for that gene in any GTeX tissue (no human auditory tissues available; each highlighted in S2 Fig) [22]. We identified two significant SNPs: rs2877561 in *ILDR1* (p = 6.2x10^-5^, Table 3, Fig 3C; Bonferroni α level for synonymous changes 9.3x10^-5^) and rs9493627 in *EYA4* (p = 0.00011, Table 3, Fig 3D; Bonferroni α level for nonsynonymous changes 0.00025). The first SNP, rs2877561, replicated in the UK Biobank (p = 0.00057), had a non-significant but consistent effect in the other GERA groups (p = 0.42), and was significant for both GERA SRT (p = 0.00019) and SDS (p = 0.0019). The second SNP, rs9493627, replicated in the UK Biobank (p = 0.0095), the other GERA race/ethnicity groups (p = 0.0080), and was marginally significant and in the correct direction for SRT (p = 0.041) and suggestively so for SDS (p = 0.081).

**Table 3:** SNPs identified in candidate gene exon analysis. The genotype is coded for the risk increasing allele, which is the first one mentioned (e.g., A in A/C). The discovery test of ARHI in GERA non-Hispanic whites was two-sided^#^, and all replication tests were one-sided. There are no estimates for African American cases SDS/SRT, as the sample size was too small. OR, odds ratio (for case/control phenotypes); Effect, effect size estimate (for SDS/SRT phenotypes); Freq, frequency of the risk increasing allele; SRT, speech reception threshold; SDS, speech discrimination score.

		rs2877561 (A/C)–exon 2 all transcripts, synonymous, *ILDR1*				rs9493627 (A/G)–exon 11 of NM_172105 & NM_004100, exon 10 of NM_172103, missense, *EYA4*			
Phenotype	Group	Freq	OR/Effect	P	r_info_^2^	Freq	OR/Effect	P	r_info_^2^
ARHI ICD-9 case/control	GERA non-Hispanic white^#^	0.281	1.090	6.2x10^-5^	0.98	0.317	1.083	0.00011	1.00
	GERA Latino	0.347	0.930	0.16	0.99	0.313	1.217	0.0037	1.00
	GERA East Asian	0.092	1.099	0.26	0.96	0.338	1.009	0.46	1.00
	GERA African American	0.190	1.141	0.20	0.96	0.505	1.176	0.095	1.00
	GERA Latino, East Asian, and African Americans replication meta-analysis	-	0.988	0.42	-	-	1.129	0.0080	-
ARHI case/control by self-report	UK Biobank	0.271	1.037	0.00057	0.98	0.319	1.025	0.0095	1.00
Cases SRT, modeled as sqrt(SRT), higher is worse hearing	GERA non-Hispanic white	0.301	0.089	0.0055	0.98	0.329	0.033	0.16	1.00
	GERA Latino	0.334	-0.096	0.75	0.99	0.372	-0.140	0.89	1.00
	GERA East Asian	0.070	-0.187	0.71	0.96	0.443	0.054	0.32	1.00
	GERA African American	-	-	-	0.96	-	-	-	1.00
	GERA Meta-analysis of all	-	0.077	0.012	-	-	0.021	0.24	-
Controls SRT	GERA non-Hispanic white	0.283	0.058	0.027	0.98	0.332	0.041	0.072	1.00
	GERA Latino	0.282	0.275	0.0011	0.99	0.350	-0.075	0.75	1.00
	GERA East Asian	0.094	0.189	0.11	0.96	0.312	0.157	0.056	1.00
	GERA African American	0.196	-0.279	0.93	0.96	0.546	0.137	0.27	1.00
	GERA Meta-analysis of all	-	0.076	0.0032	-	-	0.044	0.046	-
Overall SRT	GERA Meta-analysis of all	-	0.076	0.00019	-	-	0.034	0.041	-
Cases SDS, modeled as log(100-SDS+1), higher is worse hearing	GERA non-Hispanic white	0.303	0.045	0.065	0.98	0.332	-0.015	0.70	1.00
	GERA Latino	0.344	0.035	0.39	0.99	0.368	-0.209	0.98	1.00
	GERA East Asian	0.078	-0.135	0.65	0.96	0.449	0.318	0.0035	1.00
	GERA African American	-	-	-	0.96	-	-	-	1.00
	GERA Meta-analysis of all	-	0.044	0.070	-	-	-0.012	0.67	-
Controls SDS	GERA non-Hispanic white	0.284	0.048	0.027	0.98	0.330	0.062	0.0042	1.00
	GERA Latino	0.294	0.232	0.0033	0.99	0.338	-0.099	0.84	1.00
	GERA East Asian	0.097	0.054	0.37	0.96	0.315	0.019	0.42	1.00
	GERA African American	0.202	-0.091	0.73	0.96	0.542	0.214	0.14	1.00
	GERA Meta-analysis of all	-	0.059	0.0060	-	-	0.048	0.015	-
Overall SDS	GERA Meta-analysis of all	-	0.053	0.0019	-	-	0.024	0.081	-

### Age at onset

We also examined the age of onset distributions for the four identified SNPs (empirical cumulative distribution functions, S3 Fig). For three of the SNPs, there was no pattern towards earlier onset for the predisposing allele (rs4932196 p = 0.24, rs58389158 p = 0.75, rs2877561 p = 0.97); however, for SNP rs9493627 in EYA4, there was a suggestive trend (p = 0.044) towards earlier onset with the number of risk alleles.

### Heritability and variance explained by ARHI associated SNPs

The heritability explained by all of the genome-wide SNPs was estimated to be a somewhat modest 8.7% (95% CI = 2.9%-14.4%). This estimate depends on the assumed prevalence of ARHI in the cohort; we assessed the sensitivity to this assumption, finding that the heritability estimate was 10.7% (95% CI = 3.6%-17.8%) when using twice the prevalence. We note that this is below previous estimates from twin studies [12,13], though we discuss later why this may be an underestimate (see Discussion). In addition, the amount of variance explained by the four SNPs found here was very small at a combined 0.43% (rs49321 0.18%, rs58389185 0.12%, rs2877561 0.064%, rs9493627 0.056%) suggesting there are still many more loci to be found.

## Discussion

In this large cohort comprised of members of KPNC, we identified two novel SNP associations in the GERA non-Hispanic whites that showed replication in at least two of three subsequent analyses: meta-analysis of GERA Latinos, East Asians, and African Americans; independent related quantitative traits in a subset of GERA; and in the UK Biobank self-report data. Of note, we were unable to replicate any of the previously-described suggestive GWAS loci. However, we additionally found two SNPs associated with ARHI when specifically looking at variants in exons in previously-identified Mendelian hearing loss genes. Finally, we estimated only modest genome-wide heritability.

The indel rs58389158 on 22q13.1 that we identified to be associated with ARHI has a potential functional mechanism through the gene *TRIOBP*/*RP1-37E16*.*12*, a filamentous actin (F-actin) binding protein that is associated with the TRIO guanine nucleotide exchange factor and regulates actin cytoskeleton organization. Nonsense, missense, and frameshift mutations in this gene have been previously associated with recessive prelingual nonsyndromic hearing loss [23–27], and it has been shown to be expressed in 11 human tissues in the short isoform and 3 in the long isoform, including expression in the cochlea [26], where its potential role in hearing loss has been described in detail. The SNP is 5773bp 3’ of exon 7 in the long NM_001039141 isoform (24 exons, exon 7 the largest at 3319bp) and 1021bp 5’ of exon 8 (which is 115bp) of *TRIOBP* (and upstream of the other shorter two isoforms NM_007032, 14 exons, and NM_13862, 8 exons). It is highly correlated with rs5756795 (r^2^ = 0.96/0.97/0.97/0.94 in 1000 Genomes European/Admixed American/East Asian/African populations, respectively; b37 position = 38,122,122, 6,091bp from rs58389158), a missense variant in exon 7 (TTC to CTC, F [Phe] to L [Leu]) that was also genome-wide significant in GERA non-Hispanic whites (p = 2.8x10^-9^). According to SeattleSeq Annotation v138 [28], the rs5756795 variant is predicted to be not likely deleterious (PolyPhen2 probability of being damaging 0.006, predicted benign [29]; grantham score = 22, range from 5–215, higher more deleterious [30]), while the Combined Annotation Dependent Depletion (CADD) score [31] was borderline (13.5, scores greater than 10 indicate that the variant is predicted to be among the 10% most deleterious substitutions in the human genome); the variant also showed high conservation scores (PhastCons = 0.996, range from 0–1, with higher being more conserved [32]; Genomic Evolutionary Rate Profiling (GERP) score 2.89, ranges from -12.3 to 6.17, with 6.17 being most conserved [33]). There is potential that the missense variant itself may be affecting the protein produced, or the regulatory region around it may be affecting the transcript produced. While numerous mutations in TRIOBP have been associated with hearing loss, neither of these variants has been previously reported to be associated with hearing loss.

This GWAS also identified rs4932196 on chromosome 15. The mechanism through which this SNP leads to hearing loss is unclear, but 2 genes in the vicinity of rs4932196 and/or correlated with it (r^2^>0.80) could be relevant. The SNP is 52kb 3’ of gene *ISG20*, and 685kb 5’ of *ACAN*, which codes for Aggrecan. Aggrecan is a major component of the extracellular matrix of cartilaginous tissues while the *ISG20* protein is involved in pathways such as interferon signaling.

To identify potential mechanisms in humans of rs4932196, we assessed human tissue expression results from the ENCODE project through Haploreg v4.1 [34], which includes many different human tissue types, but no auditory tissues. The database showed that the SNP (or those highly correlated with it) lies in a region that is transcriptionally active; it is within DNAse hypersensitivity sites that are transcriptionally active in 8 tissues, and it affects the binding motifs for 38 transcription factors. The database also suggested that the SNP may be in a regulatory region–it is within enhancer histone marks for 16 different tissues. Lastly, the database showed that the SNP likely affects *ISG20* or *ACAN* expression. The expression Quantitative Trait Locus (eQTL) analysis, which tests for an association of a SNP with the expression of genes, showed that rs4932196 and the SNPs highly correlated around it are associated with expression of both *ISG20* (p ranges from 0.0013 to 6.7x10^-8^ from whole blood) and *ACAN* (p ranges from 2.9x10^-5^ to 9.2x10^-6^ in GTex 2015 cells transformed from fibroblasts, a cell type that synthesizes the extracellular matrix and collagen); Haploreg includes eQTL results from GTex, GUEVADIS, and 10 other studies. These results do not indicate whether *ISG20* or *ACAN* is the more likely mechanistic explanation for the association with ARHI.

Since human auditory tissue data were unavailable, we looked at the expression of the two genes in mouse auditory tissue using the Shared Harvard Inner Ear Database (SHIELD) [35]. Data from mice show that *ACAN* is expressed in mouse auditory tissue. Scheffer et al. looked at the expression of the genes in the cochlea and utricle at several mouse developmental stages [36]. The highest estimated expression was in the P7 developmental stage in the cochlear non-hair cells. The overall expression in the cochlear hair cells was significantly different from that in the non-hair cells (FDR = 0.00012, fold change = 0.03, S4 Fig). *ACAN* expression was higher in the cochlea than in the utricle, but the difference was not significant (FDR = 0.37, fold change = 6.2). Also, postnatal expression was higher (but not significantly) than embryonic expression (FDR = 0.31, fold change = 6.7). Liu et al. tested for gene expression differences between cochlear outer and inner hair cells in P25 to P30 mice and did not find a significant difference for *ACAN* (FDR = 0.948, fold change = 0.99) [37]. Lastly, Shin et al. tested for differential expression between the spiral and vestibular ganglia at several developmental times [38] and found no evidence for any expression differences (S4 Fig).

Mouse studies also support a role for *ISG20* in mouse auditory tissue. Expression results for the *ISG20* gene were similar to those for *ACAN*, except that the strongest expression was estimated to be in the utricle (S4 Fig). There were no significant expression differences between hair cells and non-hair cells (FDR = 0.15, fold change = 0.17), cochlea and utricle (FDR = 0.35, fold change = 0.21, utricle higher than cochlea), postnatal and embryonic (FDR = 0.51, fold change = 4.63), or cochlear outer and inner hair cells (FDR = 0.56, fold change 1.2). Finally, spiral and vestibular ganglia showed no significant differential expression of *ISG20*. The mouse expression results provide perhaps slightly more support for *ACAN* than *ISG20* as the gene near rs4932196 that is associated with hearing loss.

Because of our *TRIOBP* results, we also looked at known Mendelian hearing loss genes, particularly the exons, and discovered two SNPs, which also had evidence of replication. The first SNP, rs2877561 is a synonymous mutation in exon 2 in *ILDR1*, a gene encoding an immunoglobulin-like domain containing protein. Previous work has found 10 different homozygous mutations in the gene that cause autosomal recessive prelingual nonsyndromic moderate to profound hearing loss, that was more pronounced at higher frequencies; one of these includes a 35bp deletion at the exon 2 splice acceptor site [39,40]. The *ILDR1* gene was also shown to be expressed in the cochlea in mice, at lower to intermediate levels in hair cells, and higher levels in some supporting cells [39]. The SNP rs2877561 had high conservation scores for a nucleotide change (PhastCons 0.996, GREP 3.650, see Discussion of these scores above), but a very low CADD score (0.383, see Discussion above). The second SNP, rs9493627, is a missense mutation (GGC to AGC, G [Gly] to S [Ser]) at position 25 in exon 11 of *EYA4*, a gene that encodes a transcriptional activator, interacting with other protein families to regulate early development. Previous mutations in the gene have been shown to cause postlingual progressive autosomal dominant hearing loss, resulting in stable flat sensorineural deafness without the influence of presbycusis [41]. Two mutations causing premature stop codons were found in exon 12 with a single individual having a mutation in exon 11 at position 1270, very close to rs9493627 at position 1287. The variant rs9493627 itself had no Polyphen evidence of being damaging (0.969), had high conservation scores (PhastCons 1.000 and GERP score 5.490), a Grantham score of 56, and a CADD score of 35.0 (see Discussion of these scores above).

Our discovery of these two additional SNPs in exons of known hearing loss genes suggests potential regions of the genome to focus on for finding additional SNPs associated with ARHI. It also provides more evidence suggesting a potentially similar etiology for ARHI as hearing loss.

The use of a large cohort with comprehensive, longitudinal EHRs provided a much larger sample size than has been previously analyzed for ARHI, albeit with a slightly different phenotype than those based on hearing frequency thresholds. The advantage of such an approach is the large numbers of individuals that can be analyzed, while the disadvantage is that the cohort is not specifically characterized for hearing loss, but has to be inferred indirectly. Because of late and insidious onset of age-related hearing loss, and the fact that our primary analysis was based on ICD9 diagnosis, we expect that the ARHI effect size estimates may be biased downward and the power to detect novel loci diminished due to misclassification of individuals who did not seek treatment. It likely also dampened our genome-wide heritability estimate. However, to support the validity of our phenotype, we did see large differences in the quantitative measures SRT and SDS between cases and controls. Unfortunately we did not have data on noise exposure except for what was available in the audiologist notes in the reduced cohort–again, another limitation of a dataset not specifically designed to capture data regarding hearing loss.

The degree to which the two genome-wide significant SNPs were replicated in the other GERA race/ethnicity groups, the other GERA quantitative phenotypes, and the UK Biobank data, varied. Reasons for weaker associations in the other GERA race/ethnicity groups include both different LD structures as well as a much smaller sample size. The latter is also a reason for reduced power of analysis of the related quantitative GERA phenotypes. Finally, the UK Biobank was based on a self-reported phenotype largely at one cross-sectional survey, as opposed to our diagnosis that required having two ICD-9 diagnostic codes and an age at diagnosis. In addition, the UK Biobank individuals were at least a decade younger, on average, yet, surprisingly, had a higher proportion of cases, also indicating some inconsistency between the UK Biobank and KP phenotypes.

Our study did not replicate any previously reported sub-genome-wide significant findings seen in prior GWAS studies; this could be due to power or slightly different phenotypes, or potentially because these previously reported analyses were based on sub-genome-wide-significant loci rather than genome-wide significant loci. Although the Glutamate Receptor Metabotropic 7 (GRM7) locus has shown some replication [3], it has failed to do so in more recent results [14]. Our ARHI phenotype was also slightly different from the quantitative phenotypes used in most of these studies, which were calculated from principal components of several hearing frequency thresholds (i.e., the minimum loudness at which a hearing frequency can be heard, repeated for several frequencies, which constitute the points shown on an audiogram).

In summary, we have utilized a general cohort with a large number of middle-aged and older individuals and clinical data from EHRs to discover four loci associated with ARHI. One of these SNPs may be a missense variant in a known hearing loss gene. The second is close to two potential candidate genes through which it may exert its effect. The other two SNPs were exon variants discovered in known hearing loss genes. Studies are ongoing on these SNPS to determine how they interact with their respective surrounding candidate genes. Studies of other large genotyped cohorts with searchable EHR records, as well as the use of more precise phenotypes (e.g., scanned audiograms on a subset of the cohort) will continue to improve our understanding of the genetics underlying ARHI.

## Materials and Methods

### Participants and phenotype

Our primary analysis used non-Hispanic white individuals from the RPGEH GERA cohort, which has been previously described [18,19]. Hearing tests are part of routine care in the Kaiser Permanente health care system, and the majority of audiologist record notes are stored in the EHR, although some scan in handwritten notes. The audiograms themselves are not coded directly into the EHR numerically, and instead may be scanned images. For the current analysis, our primary phenotype was constructed by querying the EHR for ARHI related phenotypes from 01/1996-12/2014. A total of 16,123 unique individuals had at least 1 diagnosis of the ICD-9 codes 388.01 (presbycusis, 137 recorded diagnoses on 107 unique individuals), 389.12 (bilateral neural hearing loss, 135 recorded diagnoses on 63 unique individuals), and 389.19 (bilateral sensorineural hearing loss, 52,711 recorded diagnoses on 16,045 unique individuals). To help ensure the validity of cases and eliminate any potential errors in the EHR, we required ARHI cases to have at least 2 ICD-9 entries of any of these three codes, resulting in 8,285 individuals. We also ran our GWAS with a phenotype definition of at least one diagnosis; this slightly dampened our results. After excluding a small number of individuals with any single ICD-9 code for ear damage of transient ischemic deafness (388.02), noise effects on inner ear unspecified (388.10), acoustic trauma (explosive) to ear (388.11), noise-induced hearing loss (388.12), sudden hearing loss unspecified (388.2), and abnormal auditory perception unspecified (388.40), 8,111 individuals remained. For the controls, we began with the 86,790 individuals who were free of any of the ICD-9 codes used to identify cases. We further excluded individuals with any single ICD-9 368.XX (other disorders of the ear) or other 388.XX (hearing loss) code, resulting in 61,811 individuals. After excluding those who had a Current Procedural Terminology (CPT) code for hearing aids, 61,689 individuals remained. Finally, after excluding at random genetic first-degree relatives identified using King robust [42], we were left with a total of 7,569/58,652 cases/controls in GERA. We used the 6,527/45,882 GERA non-Hispanic white cases/controls in our discovery cohort. Note that we also required 2 or more ICD-9 codes for diagnosis of the phenotypes we used as covariates–hypertension 401.XX and 997.91, osteoporosis 733.XX, and diabetes 250.XX, traits which have been shown to be potentially associated with ARHI.

We also examined a secondary set of hearing related phenotypes on a much smaller subset of the cohort that had audiologist notes in the EHR data (the majority from cases). Speech Discrimination Scores (SDS), or the percentage of words that can be recognized when speech is loud enough to be heard comfortably, and Speech Recognition Threshold (SRT) measurements, or the decibel level at which an individual can understand 50% of spoken words tested, were available on 4,903 unrelated GERA individuals (9,454 total measurements, as some individuals had multiple independent measurements at different times) whose EHR included notes taken by their audiologist. Most electronic notes followed a specific template, making extracting certain phenotype information easier. For our outcome, we extracted both SDS and SRT scores, averaging the left and right ear scores. We also attempted to quantify a crude noise exposure history indicator variable based on recorded text from audiologist notes regarding potential noise exposure occupations and activities from these notes (only available on the same subset, so only SDS and SRT were adjusted for noise). To identify potential noise exposure words that had been recorded in each individual’s notes, we began by constructing an exhaustive list of all possible words that were in these notes. We identified a total of 31,825 unique words from the combined set of all individuals’ notes. This was a short enough list that we could simply manually inspect each of these unique words to determine all noise exposure variables given by patients. Once all possible noise exposure variables were identified, we searched for either presence of the noise variable vs. absence of each of the noise exposure variables in the text (e.g., patient denies vs. patient reports). If a noise variable was identified, then that individual was assumed to have a history of noise exposure. If noise variables were absent in the audiologist notes, we assumed the individual had no history of noise exposure.

The Kaiser Permanente Northern California Institutional Review Board (study #CN-13-1643-H) and the University of California San Francisco Human Research Protection Program Committee on Human Research (study #13–12476) approved this research project. All participants provided written informed consent.

### Genotyping, quality control, and imputation

A total of 110,266 GERA cohort individuals were genotyped on one of four race/ethnicity-specific Affymetrix Axiom arrays optimized for individuals of European (EUR), Latino (LAT), East Asian (EAS), and African American (AFR) race/ethnicity [43,44]. Quality control was performed on an array-wise basis, which has been described [19]. Briefly, 102,998 individuals passed QC in the first pass genotyping round with individual DishQC (DQC)>82% and individual CR>97%. A total of 85 individuals failed X chromosome heterozygosity tests (male≤20%, female≥80%), leaving 102,913 individuals (step added not in [19]). Genotypes were filtered by package (plates of 96 samples were grouped by similar assay conditions into 58 packages), retaining SNPs with CR≥90%, variance ratio≥31, and male/female frequency differences≤15% [19]. SNPs with poor duplicate concordance were removed, and those with overall call rate<60% were removed, as described in [19]. Here, we additionally required a stricter array per-SNP call-rate of 90%, resulting in 665,046 (EUR), 775,597 (LAT), 702,405 (EAS), and 863,961 (AFR) SNPs, respectively. Finally, to avoid SNPs with low minor allele counts within each race/ethnicity group, we excluded SNPs with a minor allele frequency (MAF) less than 0.0015 (EUR), 0.02 (LAT), 0.025 (EAS), and 0.065 (AFR), leaving a total number of SNPS of 659,803 (EUR), 648,979 (LAT), 588,493 (EAS), 543,158 (AFR), respectively, and a total of 1,188,134 unique genotyped SNPs available for analysis across all race/ethnicity groups.

Imputation was also performed on an array-wise basis, and before the MAF cutoff described above was applied. Genotypes were first pre-phased with Shape-it v2.r72719 [45]. We then imputed 31,085,734 variants from the 1000 Genomes Project (phase I integrated release, March 2012, with Aug 2012 chromosome X update, with singletons removed) as a cosmopolitan reference panel with Impute2 v2.30 [46–48]. The estimated quality control metric used in this study, r_info_^2^, is the info metric from Impute2, which gives an estimate of the correlation of the imputed genotype to the true genotype [49]. SNPs that did not impute with high quality (r_info_^2^<0.8) were removed. The r_info_^2^ and MAF exclusion criteria for the imputed SNPs resulted in a final number of SNPs of 9,469,183 (EUR), 8,090,486 (LAT), 6,517,021 (EAS), and 7,829,026 (AFR), and a total of 11,910,003 unique imputed SNPs available for analysis across all race/ethnicity groups.

### GWAS analysis and covariate adjustment

For our discovery cohort primary analysis, we used the GERA non-Hispanic whites, employing the conventional genome-wide significance level of 5x10^-8^. Data from each SNP was modeled using additive dosages, which account for the uncertainty in imputation [50]. We ran a logistic regression on ARHI diagnosis, including covariates for age (at diagnosis for cases, and at last follow-up for controls), diabetes, hypertension, and osteoporosis, and also included sex and the first ten ancestry PCs for non-Hispanic whites to adjust for genetic ancestry/population stratification; the PC analysis has been previously described [18]. For computational efficiency we first ran a logistic model including all covariates except the SNP, and collapsed them into a single covariate for each individual as the sum of the coefficients times each of the covariates for each individual, and then ran a logistic model including only the SNP and the collapsed covariate. Including a single covariate with the SNP genotype reduced the computing time, without loss of accuracy because the SNP effects are quite modest and have virtually no influence on the other covariates. We further confirmed that our top hits showed no appreciable difference fit this way versus the full model (the odds ratio estimates were identical).

For the secondary phenotypes available on a subset of the cohort, we transformed the quantitative phenotypes by taking the square root of SRT, and log(100—SDS + 1) to make them more normally distributed. We ran a mixed model regression analysis on each of the transformed SDS and transformed SRT values (to account for repeated measures), including the same covariates as described above in the analysis of ARHI, as well as noise exposure. We analyzed the transformed SDS and SRT phenotypes separately in ARHI cases and controls, and then meta-analyzed them, to construct a test that was independent of the ARHI test.

### Replication in GERA Latinos, East Asians, African Americans, and UK Biobank individuals

To determine whether the novel SNPs identified in GERA non-Hispanic whites constituted replicable findings, we evaluated the SNPs in 481/5,215 GERA case/control Latinos, 398/5,040 GERA East Asians, and 146/2,133 GERA African Americans (there were an additional 17 South Asian cases, too few to analyze), in addition to 30,802/78,586 self-reported case/control individuals from the initial preliminary release of the UK Biobank; complete details of this cohort are available at www.biobank.ac.uk, and the cohort has been previously described [51]. For the SRT and SDS phenotypes, there were 298 Latinos, 249 East Asians, and 125 African Americans available for analysis. For the three GERA minority groups, we used the analysis methods described above, but adjusting for the first six genetic ancestry PCs in each of the three minority groups. A strict Bonferroni α-level for two tests is 0.05/2 = 0.025. In addition, all replication tests are based on a single direction alternative hypothesis (i.e., the effect size in the same direction as in the discovery), therefore we report one-sided P-values.

Cases for the UK Biobank were identified by a self-reported yes to the question “Do you have any difficulty with your hearing?” (variable 2247.0/1/2.0) and a yes to the question “Do you find it difficult to follow a conversation if there is background noise (such as a TV, radio, children playing)” (2257.0–2.0). Individuals who reported being completely deaf were excluded. Controls were identified as those answering no to both of the previous hearing questions, as well as not reporting having a hearing aid (3393.0–2.0). Age was reported as the age at the time of survey (as opposed to age of diagnosis, which was unavailable; cases average age = 58.8, sd = 7.4; control average age = 55.6, sd = 8.1). Analysis was based on a logistic regression model including the SNP as the dependent variable and adjusting for sex (22001.0.0), hypertension (defined as average of SBP≥140 or DBP≥90; variables 93.0–2.0–1, 94.0–2.0–1, 4079.0–2.0/1, 4080.0–2.0–1), self-reported diabetes (2443.0–2.0), self-reported occupational/loud music noise exposure (4825.0–2.0, 4836.0–2.0), and 10 ancestry PCs. First-degree relatives identified by King robust [42] were randomly removed. Individuals were further restricted to those whose self-reported ancestry (21000.0–2.0) was from any white group and whose global genetic ancestry PC_1_<50 and PC_2_<50, where these PCs were calculated from the entire cohort (22009.0.1–2). This resulted in a total of 30,802 cases and 78,586 controls. We then re-calculated ancestry PCs in this group using a set of 371,038 very high quality SNPs (call rate>99%, MAF>1%, and LD filtered so no two SNPs had r^2^>0.5) and using 50,000 random individuals with the remainder projected, as described previously [18].

### Replication analysis of previously described suggestive Loci

We also tested 58 previously described sub-threshold suggestive SNPs for replication in this study. The phenotypes of three studies have been based on PCs of several hearing frequency thresholds (3,417 European ancestry individuals in [15], 2,161 Belgian individuals [14], and 352 Finnish Saami individuals [16]). We included in this analysis any SNPs reported in their supplemental material with P<1x10^-5^. We also included the GRM7 SNPs from the study of 846 cases and 846 controls [3]. For the replication p-value threshold we used 0.05 divided by the number of SNPs compared (α = 0.00086), and we also report any nominal associations (p<0.05).

### ARHI associations at previously identified hearing loss genes

We examined genetic variation at 132 known hearing loss genes for association with ARHI [21] using our GWAS results at reduced significance thresholds to reflect the proportion of the genome being tested to account for potential lack of power in these regions. For each of the genes, we looked separately at exonic SNPs, specifically non-synonymous and synonymous coding changes, and then SNPs that were eQTLs for that gene in any GTeX tissue (no human auditory tissues available) [22]. We looked ±50Kb of each gene, or up to any eQTL. This led to 414,466 imputed SNPs, or 4.7% of the genome (the usual genome-wide correction is for 1 million tests, despite more SNPs, to correct for correlation, so a Bonferroni correction for percentage of the genome would be 0.05/47000 = 1.1x10^-5^, which no SNPs met). We further looked into the 253 nonsynonymous exon changes; to determine the correction factor for multiple testing, we decomposed the correlation matrix of these SNPs (based on pairwise LD r-square values) into the M eigenvalues λ_1_, …, λ_M_, and solved for M* such that (∑_m = 1,…,M*_ λ_m_)/(∑_m = 1,…,M_ λ_m_ ≥ c, for c = 0.95 [52], which has additionally shown to perform well [53]. For the nonsynonymous changes we found that M* = 202, for a Bonferroni correction of α = 0.00025. For the 909 synonymous changes, we found that M* = 536, for a Bonferroni correction of α = 9.3x10^-5^.

### Heritability from all GWAS SNPs and variance explained by ARHI associated SNPs

We estimated the additive array heritability of ARHI using Genome-wide Complex Trait Analysis (GCTA) v1.24 [54]. Since array heritability estimates may potentially be more sensitive to artifacts than GWAS results [55], we restricted our analysis to the largest group of individuals, non-Hispanic whites who had been run on the same reagent kit and type of microarray [19], and imposed the following extra filters on the autosomal SNPs: Hardy-Weinberg equilibrium test p<0.05 (in controls), significant differences in case-control missing p<0.05, and absolute MAF differences >0.15 when compared with the 1000 Genomes Project. Finally, we also employed an LD filter so that no two neighboring pairwise SNPs had r^2^>0.8, leaving 427,157 SNPs. In all analyses we further removed individuals outside 5 standard deviations of the first two genetic ancestry PCs, and also removed individuals so that no two remaining individuals had an estimated kinship>0.025 in a manner that maximized the remaining sample size, using PLINK v1.9 [56]. The remaining sample had a total of 4,603 cases and 34,136 controls. The heritability estimate depends on the prevalence of the disease through a liability threshold model; we used the prevalence of ARHI in the non-Hispanic whites in our cohort of 12.5%, and explored several other estimates (we note this is an age-averaged threshold). In addition, we estimated the variance explained by the ARHI associated SNPs [57].

## Supporting Information

S1 Table Results at previously-identified GWAS sub-threshold SNPs in GERA. Previously-identified results were large based on hearing threshold principal components, which are not directly comparable to the results here.
(PDF)

S1 Fig Q-Q plot for GERA non-Hispanic whites. Previously-identified sub-threshold SNPs are separated from the rest of the SNPs (dark green is the SNP itself, light green is within 0.5Mb of the SNP). Typed SNPs are circles, imputed are triangles.
(PDF)

S2 Fig Results at previously-identified Mendelian hearing loss genes. Results at previously-identified Mendelian hearing loss genes. SNPs between the start and end of the gene colored in blue, non-synonymous coding changes in red, other exon next steps in orange, eQTL SNPs in green.
(PDF)

S3 Fig Age of onset distributions in non-Hispanic white cases. Age of onset distributions for the ARHI variants in GERA non-Hispanic whites, based on residuals and normalized to a female without diabetes, hypertension, or osteoporosis.
(PDF)

S4 Fig Mouse tissue expression of *ACAN* and *ISG20*. FACS, fluorescence-activated cell sorting hair cells. E represents embryonic tissue, P postnatal. Numbers represent numbers of days.
(PDF)
