# Supplementary material for: A Large Genome-Wide Association Study of Age-Related Hearing Impairment Using Electronic Health Records
Source: PLoS Genet. 2016 Oct 20;12(10):e1006371. doi: 10.1371/journal.pgen.1006371 (PMC5072625; doi:10.1371/journal.pgen.1006371)
Supplement: S1 Table — Previously-identified results were large based on hearing threshold principal components, which are not directly comparable to the results here. (PDF) [file pgen.1006371.s001.pdf]

**S1 Table. Results at previously-identified GWAS sub-threshold SNPs in GERA.** Previously-identified results were largely based on hearing threshold principal components, which are not directly comparable to the results here.

| | | | | | non-Hispanic white | | Latino | | East Asian | | African American | | Meta-analysis | | |
|---|---|---|---|---|---|---|---|---|---|---|---|---|---|---|---|
| Study | SNP | Chr | Pos | Allele | HR | P | HR | P | HR | P | HR | P | HR | P | $I^2$ |
| Girotto et al., PC2 | rs6669265 | 1 | 27361219 | A/G | 1.016 | 0.47 | 0.923 | 0.33 | 1.259 | 0.23 | 1.099 | 0.62 | 1.013 | 0.53 | 0.0 |
| Girotto et al., PC3 | rs621215 | 1 | 99619424 | A/G | 0.994 | 0.76 | 0.989 | 0.88 | 0.908 | 0.27 | 1.080 | 0.54 | 0.991 | 0.64 | 0.0 |
| Girotto et al., PC3 | rs712932 | 1 | 99651500 | C/G | 0.982 | 0.4 | 0.913 | 0.22 | 0.898 | 0.22 | 1.266 | 0.06 | 0.979 | 0.28 | 50.7 |
| Girotto et al., PC3 | rs712931 | 1 | 99654168 | T/G | 0.982 | 0.4 | 0.913 | 0.22 | 0.898 | 0.22 | 1.266 | 0.06 | 0.979 | 0.28 | 50.7 |
| Girotto et al., PC3 | rs11485142 | 1 | 218833214 | C/A | 0.992 | 0.71 | 0.922 | 0.36 | 0.789 | 0.54 | 0.791 | 0.17 | 0.983 | 0.43 | 0.0 |
| Girotto et al., PC1 | rs6742663 | 2 | 20395160 | G/A | 1.021 | 0.35 | 1.073 | 0.34 | 1.030 | 0.75 | 1.185 | 0.18 | 1.029 | 0.16 | 0.0 |
| Girotto et al., PC1 | rs6544887 | 2 | 46577212 | C/T | 0.993 | 0.7 | 1.023 | 0.75 | 0.860 | 0.15 | 0.962 | 0.78 | 0.990 | 0.57 | 0.0 |
| Girotto et al., PC1 | rs6544889 | 2 | 46577299 | A/G | 0.991 | 0.65 | 1.026 | 0.73 | 0.889 | 0.27 | 0.915 | 0.56 | 0.989 | 0.54 | 0.0 |
| Girotto et al., PC2 | rs17864603 | 2 | 51989400 | C/T | 1.008 | 0.94 | 1.101 | 0.82 | 0.000 | 0.45 | 1.902 | 0.6 | 1.018 | 0.86 | 0.0 |
| Girotto et al., PC3 | rs6756828 | 2 | 174919189 | T/C | 1.012 | 0.59 | 0.968 | 0.67 | 0.921 | 0.32 | 0.994 | 0.97 | 1.003 | 0.89 | 0.0 |
| Friedman et al., case/control | rs11928865 | 3 | 7155702 | A/T | 1.007 | 0.76 | 0.954 | 0.61 | 0.930 | 0.48 | 1.141 | 0.33 | 1.004 | 0.85 | 0.0 |
| Friedman et al., case/control | rs779701 | 3 | 7518772 | G/A | 0.994 | 0.79 | 0.938 | 0.42 | 0.880 | 0.12 | 1.073 | 0.61 | 0.985 | 0.45 | 0.0 |
| Friedman et al., case/control | rs779706 | 3 | 7524042 | C/G | 0.995 | 0.81 | 0.977 | 0.76 | 0.874 | 0.099 | 1.201 | 0.16 | 0.990 | 0.63 | 35.1 |
| Girotto et al., PC2 | rs2276957 | 4 | 122261475 | C/T | 0.943 | 0.042 | 0.955 | 0.57 | 1.042 | 0.59 | 0.832 | 0.43 | 0.953 | 0.059 | 0.0 |
| Fransen et al., PC2 | rs1494630 | 5 | 63394170 | A/G | 0.962 | 0.35 | 1.207 | 0.3 | 1.145 | 0.83 | 1.102 | 0.58 | 0.980 | 0.61 | 0.0 |
| Van Laer et al., PC3 | rs457717 | 5 | 75920972 | G/A | 0.997 | 0.87 | 1.117 | 0.12 | 0.948 | 0.49 | 1.165 | 0.25 | 1.005 | 0.8 | 28.1 |
| Fransen et al., PC3 | rs116439186 | 6 | 29943067 | A/G | 1.024 | 0.26 | 1.186 | 0.025 | 1.273 | 0.0038 | 1.086 | 0.59 | 1.048 | 0.018 | 67.7 |
| Fransen et al., PC1 | rs528808 | 6 | 100104289 | G/A | 0.959 | 0.078 | 0.852 | 0.092 | 0.697 | 0.086 | 0.989 | 0.97 | 0.949 | 0.022 | 18.2 |
| Fransen et al., PC1 | rs6910580 | 6 | 110243292 | C/T | 0.981 | 0.34 | 1.110 | 0.15 | 1.127 | 0.15 | 0.914 | 0.5 | 0.995 | 0.77 | 45.4 |
| Fransen et al., PC1 | rs78236468 | 6 | 121088770 | G/T | 1.009 | 0.84 | 0.847 | 0.19 | 0.990 | 0.94 | 0.474 | 0.25 | 0.987 | 0.74 | 0.4 |
| Van Laer et al., PC2 | rs10499138 | 6 | 128492182 | T/A | 1.524 | 0.051 | 2.819 | 0.0014 | 1.184 | 0.33 | 0.646 | 0.18 | 1.321 | 0.017 | 72.9 |
| Fransen et al., PC1 | rs7786410 | 7 | 55922323 | G/A | 0.974 | 0.44 | 1.069 | 0.46 | 1.007 | 0.93 | 0.942 | 0.67 | 0.986 | 0.63 | 0.0 |
| Fransen et al., PC3 | rs62459614 | 7 | 70587145 | C/T | 0.982 | 0.8 | 1.186 | 0.094 | 0.969 | 0.69 | 1.016 | 0.96 | 1.018 | 0.7 | 0.0 |
| Fransen et al., PC3 | rs62459619 | 7 | 70598935 | T/C | 0.993 | 0.92 | 1.181 | 0.099 | 0.951 | 0.54 | 0.899 | 0.76 | 1.015 | 0.76 | 3.8 |
| Girotto et al., PC1 | rs2687481 | 7 | 125869122 | T/G | 1.023 | 0.36 | 0.995 | 0.95 | 0.966 | 0.7 | 0.996 | 0.97 | 1.017 | 0.46 | 0.0 |
| Fransen et al., PC3 | rs17170356 | 7 | 146887868 | G/A | 1.103 | 0.037 | 0.787 | 0.23 | 0.950 | 0.64 | 0.866 | 0.7 | 1.060 | 0.17 | 29.1 |
| Fransen et al., PC1 | rs7816613 | 8 | 102729475 | C/T | 0.982 | 0.55 | 1.125 | 0.3 | 0.965 | 0.8 | 1.078 | 0.62 | 0.993 | 0.8 | 0.0 |
| Fransen et al., PC1 | rs1460237 | 8 | 105841855 | A/T | 0.978 | 0.75 | 0.883 | 0.61 | 0.905 | 0.92 | 3.519 | 0.37 | 0.974 | 0.68 | 0.0 |
| Girotto et al., PC3 | rs305256 | 8 | 137568252 | C/T | 1.002 | 0.94 | 1.001 | 0.99 | 1.070 | 0.47 | 1.040 | 0.81 | 1.006 | 0.78 | 0.0 |
| Girotto et al., PC2 | rs17292288 | 9 | 129058500 | T/C | 1.001 | 0.98 | 1.070 | 0.42 | 0.840 | 0.1 | 0.699 | 0.05 | 0.993 | 0.75 | 57.4 |
| Fransen et al., PC1 | rs9329289 | 10 | 2542389 | G/A | 0.996 | 0.82 | 1.025 | 0.73 | 1.258 | 0.046 | 1.127 | 0.36 | 1.007 | 0.72 | 38.7 |
| Girotto et al., PC1 | rs6585804 | 10 | 123980108 | A/G | 0.965 | 0.073 | 1.089 | 0.25 | 0.852 | 0.14 | 1.049 | 0.71 | 0.971 | 0.11 | 30.8 |
| Girotto et al., PC1 | rs585747 | 11 | 79700145 | G/A | 0.992 | 0.7 | 0.859 | 0.037 | 1.044 | 0.6 | 1.062 | 0.64 | 0.987 | 0.48 | 33.3 |
| Girotto et al., PC3 | rs7123014 | 11 | 85922442 | A/G | 0.993 | 0.71 | 0.934 | 0.34 | 1.179 | 0.032 | 1.023 | 0.87 | 0.999 | 0.96 | 46.9 |
| Girotto et al., PC1 | rs3765620 | 11 | 102595492 | A/G | 1.020 | 0.3 | 1.008 | 0.91 | 0.987 | 0.87 | 1.113 | 0.46 | 1.019 | 0.29 | 0.0 |
| Girotto et al., PC2 | rs11219981 | 11 | 125109627 | A/G | 0.968 | 0.16 | 0.980 | 0.84 | 0.949 | 0.57 | 0.911 | 0.72 | 0.967 | 0.13 | 0.0 |
| Fransen et al., PC2 | rs10876222 | 12 | 52362479 | A/T | 0.967 | 0.13 | 1.001 | 0.99 | 0.891 | 0.17 | 1.122 | 0.41 | 0.968 | 0.11 | 0.0 |
| Fransen et al., PC2 | rs2252518 | 12 | 52381026 | A/C | 0.964 | 0.097 | 0.995 | 0.94 | 0.902 | 0.22 | 1.134 | 0.33 | 0.966 | 0.094 | 0.0 |
| Girotto et al., PC1 | rs10848114 | 12 | 130979355 | T/C | 0.999 | 0.97 | 1.055 | 0.45 | 1.088 | 0.34 | 0.937 | 0.61 | 1.006 | 0.76 | 0.0 |
| Girotto et al., PC2 | rs11842701 | 13 | 60431689 | A/T | 1.105 | 0.22 | 1.395 | 0.33 | 0.895 | 0.48 | 0.365 | 0.38 | 1.065 | 0.37 | 0.0 |
| Girotto et al., PC2 | rs3783041 | 13 | 60588891 | A/T | 1.096 | 0.26 | 1.373 | 0.35 | 0.870 | 0.36 | 0.803 | 0.84 | 1.053 | 0.46 | 0.0 |
| Girotto et al., PC1 | rs17687720 | 13 | 102611999 | C/A | 1.057 | 0.084 | 0.968 | 0.72 | 0.814 | 0.13 | 0.923 | 0.73 | 1.033 | 0.27 | 29.8 |
| Girotto et al., PC3 | rs10130121 | 14 | 36624751 | T/G | 1.055 | 0.11 | 0.841 | 0.24 | 2.355 | 0.049 | 0.887 | 0.54 | 1.043 | 0.19 | 53.4 |
| Girotto et al., PC3 | rs17461837 | 14 | 36644330 | T/C | 1.057 | 0.1 | 0.823 | 0.19 | 2.424 | 0.037 | 0.903 | 0.6 | 1.045 | 0.18 | 57.9 |
| Girotto et al., PC1 | rs978091 | 14 | 43344558 | G/A | 1.039 | 0.17 | 1.009 | 0.91 | 1.074 | 0.42 | 1.168 | 0.22 | 1.044 | 0.084 | 0.0 |
| Girotto et al., PC2 | rs1952587 | 14 | 57161217 | A/T | 1.020 | 0.35 | 1.092 | 0.23 | 0.963 | 0.64 | 0.673 | 0.015 | 1.015 | 0.44 | 62.0 |
| Girotto et al., PC2 | rs928108 | 14 | 57170474 | G/A | 1.038 | 0.079 | 1.022 | 0.75 | 0.859 | 0.052 | 0.753 | 0.077 | 1.020 | 0.32 | 67.0 |
| Girotto et al., PC1 | rs7182802 | 15 | 27347206 | T/C | 1.010 | 0.67 | 1.043 | 0.63 | 1.036 | 0.65 | 1.167 | 0.27 | 1.018 | 0.42 | 0.0 |
| Girotto et al., PC1 | rs17705835 | 15 | 93662283 | T/C | 1.028 | 0.47 | 0.894 | 0.41 | 1.203 | 0.055 | 1.905 | 0.05 | 1.047 | 0.19 | 57.0 |
| Girotto et al., PC2 | rs7212433 | 17 | 30083687 | C/T | 1.002 | 0.9 | 1.055 | 0.47 | 0.950 | 0.62 | 0.998 | 0.99 | 1.004 | 0.83 | 0.0 |
| Girotto et al., PC1 | rs1036987 | 17 | 74892171 | T/A | 1.016 | 0.69 | 0.859 | 0.14 | 1.108 | 0.43 | 1.050 | 0.73 | 1.006 | 0.87 | 0.4 |
| Fransen et al., PC3 | rs12955474 | 18 | 57152038 | C/T | 0.982 | 0.66 | 0.999 | 0.99 | 1.035 | 0.66 | 0.799 | 0.45 | 0.991 | 0.8 | 0.0 |
| Girotto et al., PC3 | rs594821 | 18 | 76745589 | T/C | 1.013 | 0.74 | 0.956 | 0.76 | 1.400 | 0.015 | 0.755 | 0.14 | 1.021 | 0.56 | 61.8 |
| Fransen et al., PC1 | rs55655041 | 19 | 30995200 | C/T | 1.011 | 0.69 | 0.941 | 0.6 | 0.929 | 0.81 | 1.190 | 0.37 | 1.009 | 0.71 | 0.0 |
| Girotto et al., PC1 | rs11671149 | 19 | 54170468 | C/T | 0.917 | 0.34 | 1.652 | 0.058 | 4.248 | 0.039 | 1.525 | 0.6 | 1.002 | 0.98 | 66.9 |
| Fransen et al., PC2 | rs16989760 | 22 | 31951808 | G/A | 0.994 | 0.88 | 0.923 | 0.7 | 0.807 | 0.55 | 0.739 | 0.29 | 0.982 | 0.66 | 0.0 |
| Fransen et al., PC2 | rs62240516 | 22 | 32135582 | C/T | 0.994 | 0.92 | 1.238 | 0.36 | 0.895 | 0.83 | 0.903 | 0.87 | 1.004 | 0.95 | 0.0 |
| Fransen et al., PC2 | rs62238885 | 22 | 32138138 | A/G | 0.994 | 0.91 | 1.248 | 0.34 | 0.894 | 0.83 | 0.805 | 0.73 | 1.003 | 0.95 | 0.0 |
