# Supplementary material for: A Large Genome-Wide Association Study of Age-Related Hearing Impairment Using Electronic Health Records
Source: PLoS Genet. 2016 Oct 20;12(10):e1006371. doi: 10.1371/journal.pgen.1006371 (PMC5072625; doi:10.1371/journal.pgen.1006371)

S1 Fig. QQ-plot for GERA non-Hispanic whites. Previously-identified sub-threshold SNPs are separated from the rest of the SNPs (dark green is the SNP itself, light green is within 0.5Mb of the SNP). Typed SNPs are circles, imputed are triangles.

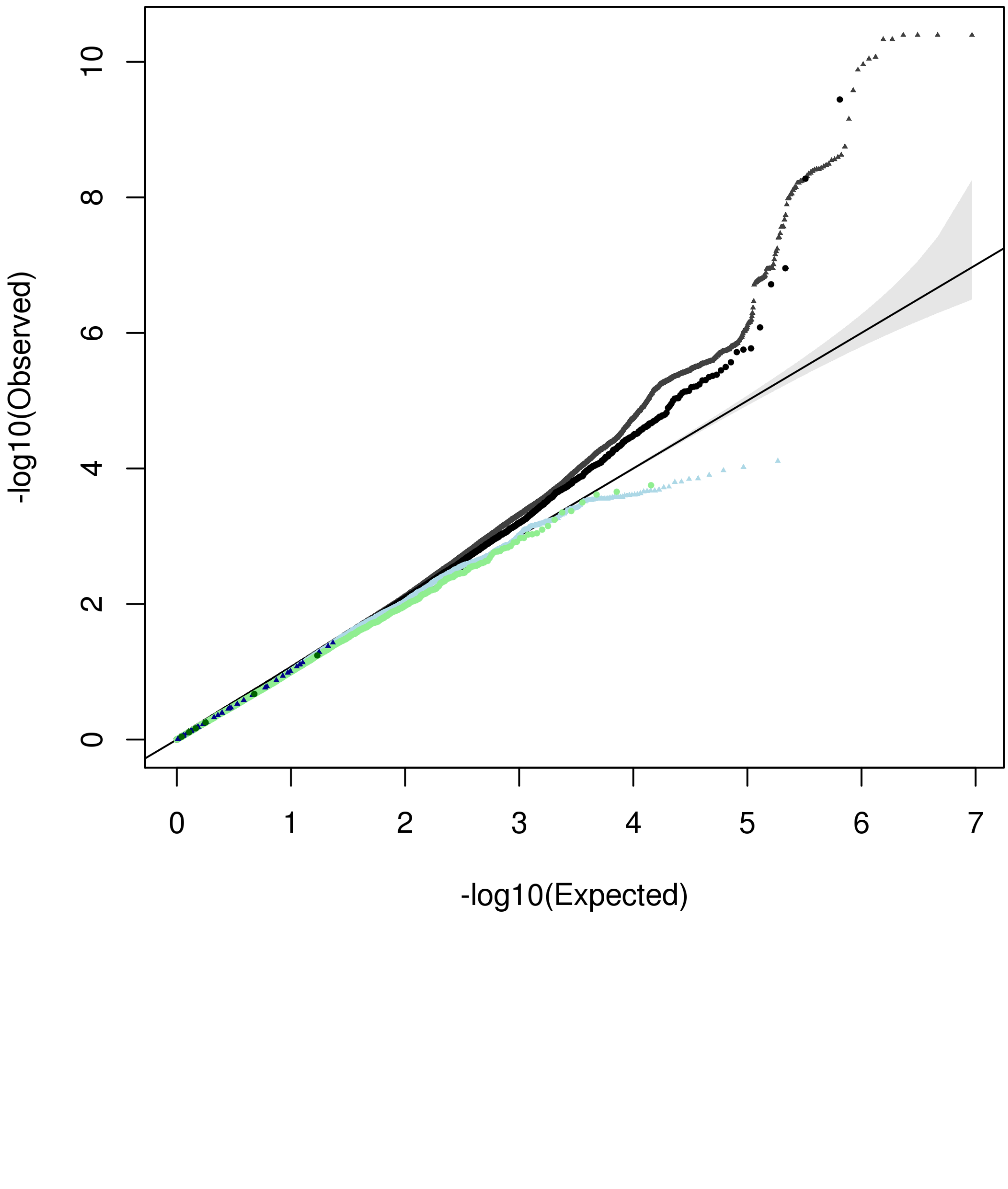

Supplement: S1 Fig — Previously-identified sub-threshold SNPs are separated from the rest of the SNPs (dark green is the SNP itself, light green is within 0.5Mb of the SNP). Typed SNPs are circles, imputed are triangles. (PDF) [file pgen.1006371.s002.pdf]
