# Supplementary material for: A Large Genome-Wide Association Study of Age-Related Hearing Impairment Using Electronic Health Records
Source: PLoS Genet. 2016 Oct 20;12(10):e1006371. doi: 10.1371/journal.pgen.1006371 (PMC5072625; doi:10.1371/journal.pgen.1006371)
Supplement: S2 Fig — Results at previously-identified Mendelian hearing loss genes. SNPs between the start and end of the gene colored in blue, non-synonymous coding changes in red, other exon next steps in orange, eQTL SNPs in green. (PDF) [file pgen.1006371.s003.pdf]

S2 Fig. Results at previously-identified Mendelian hearing loss genes. SNPs between the start and end of the gene colored in blue, nonsynonymous coding changes in red, other exonic SNPs in orange, eQTL SNPs in green.

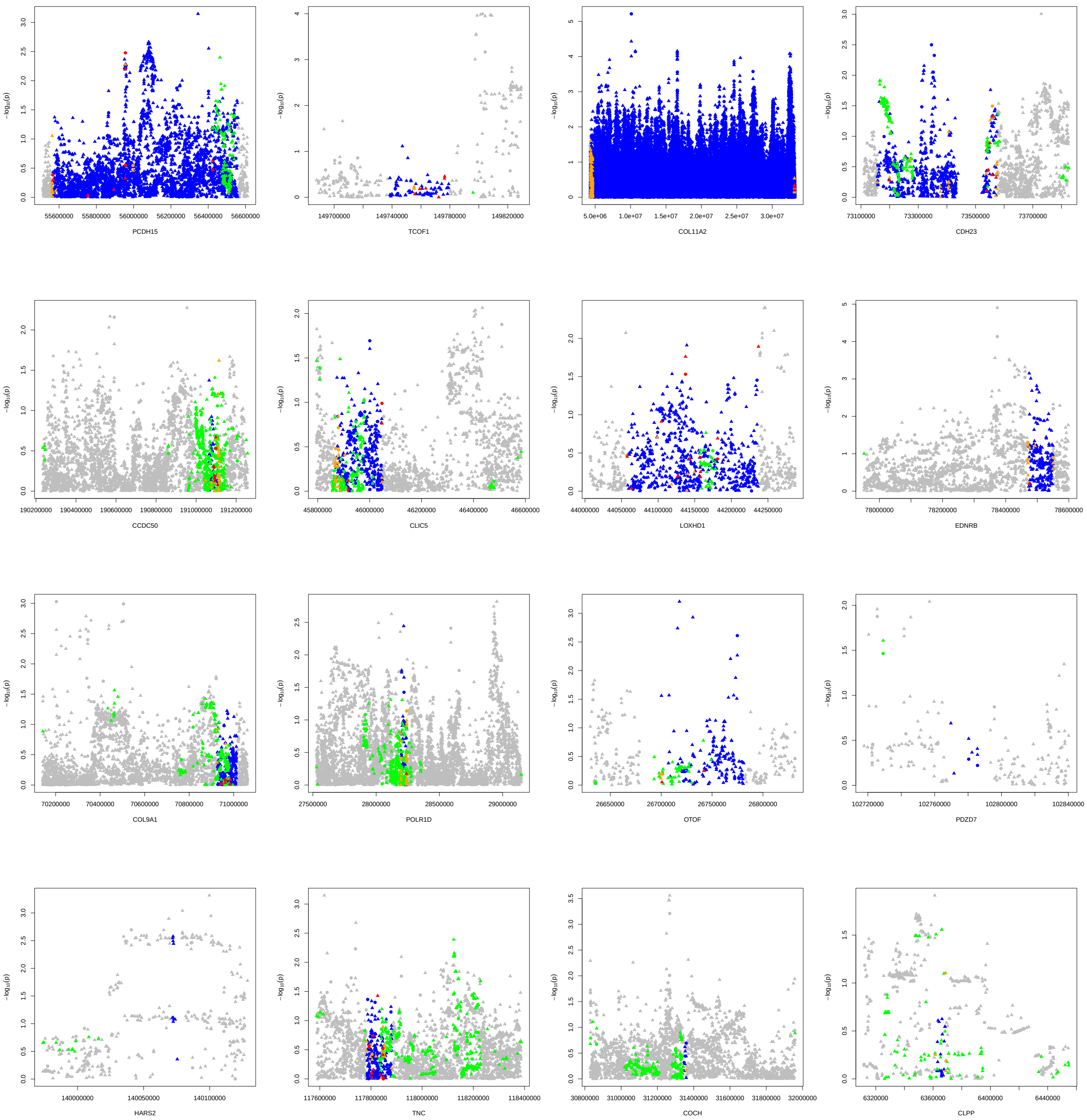

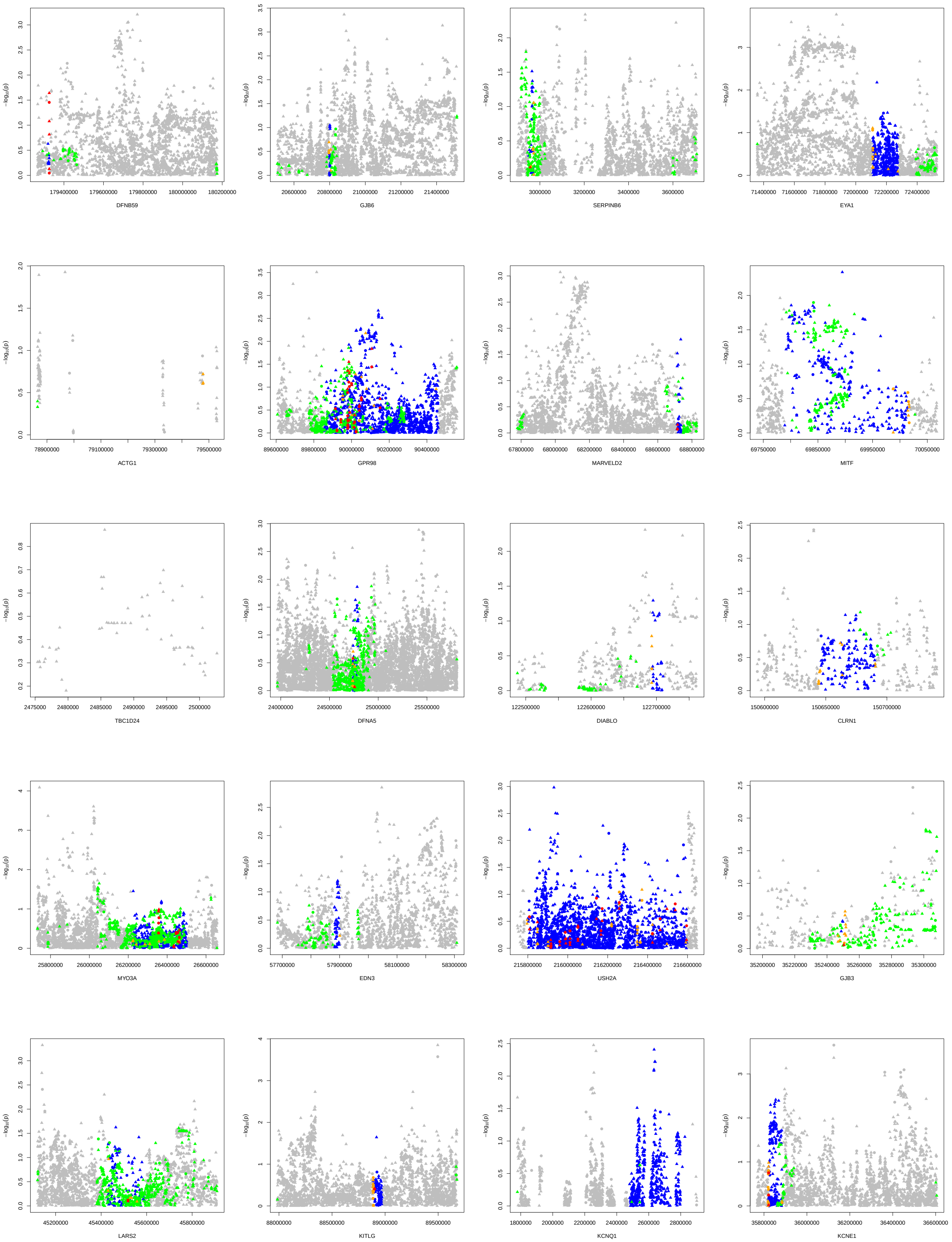
−log10(p)
DFNB59
GJB6
SERPINB6
EYA1
ACTG1
GPR98
MARVELD2
MITF
TBC1D24
DFNA5
DIABLO
CLRN1
MYO3A
EDN3
USH2A
GJB3
LARS2
KITLG
KCNQ1
KCNE1

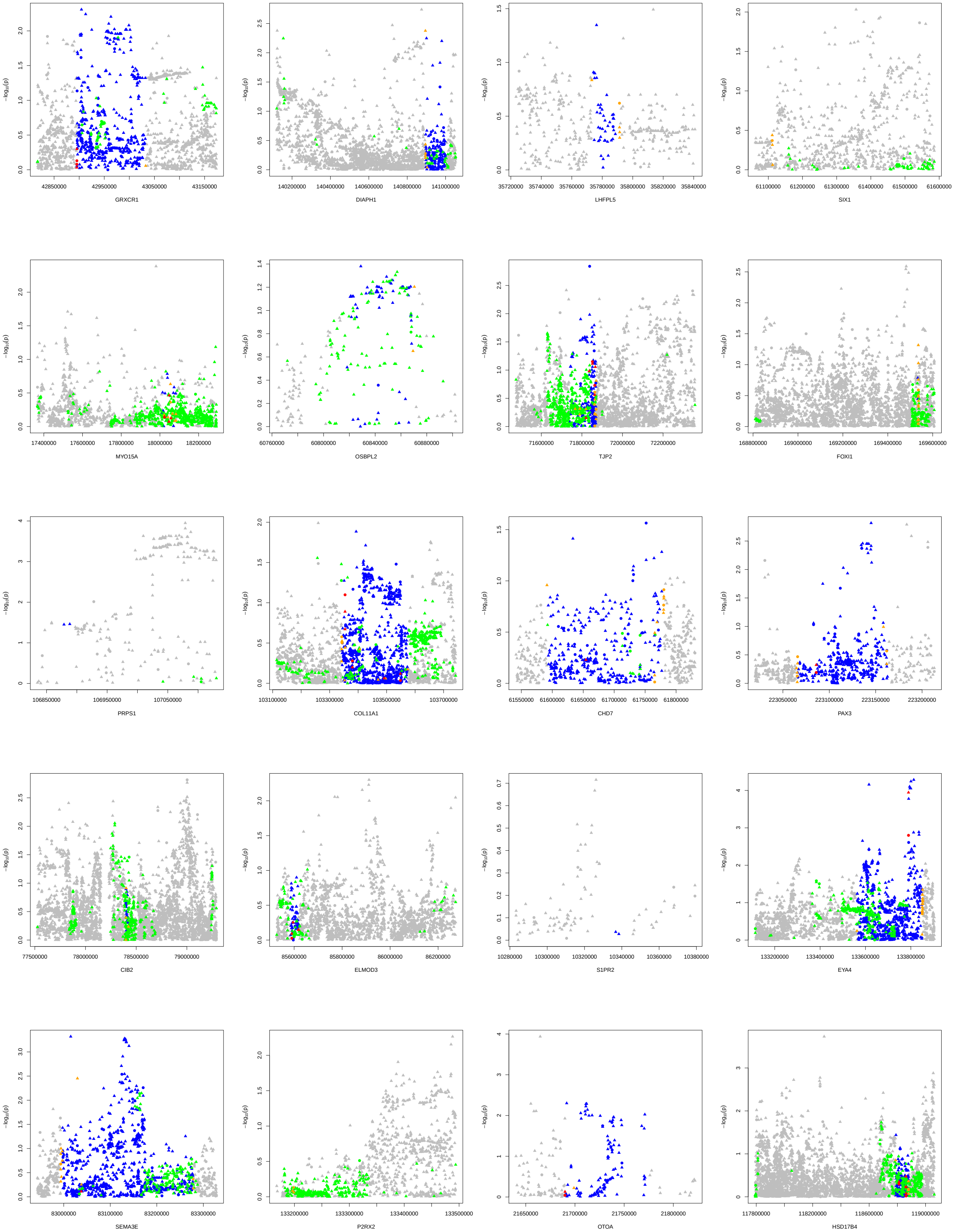
GRXCR1
DIAPH1
LHFPL5
SIX1
MYO15A
OSBPL2
TJP2
FOXI1
PRPS1
COL11A1
CHD7
PAX3
CIB2
ELMOD3
S1PR2
EYA4
SEMA3E
P2RX2
OTOA
HSD17B4

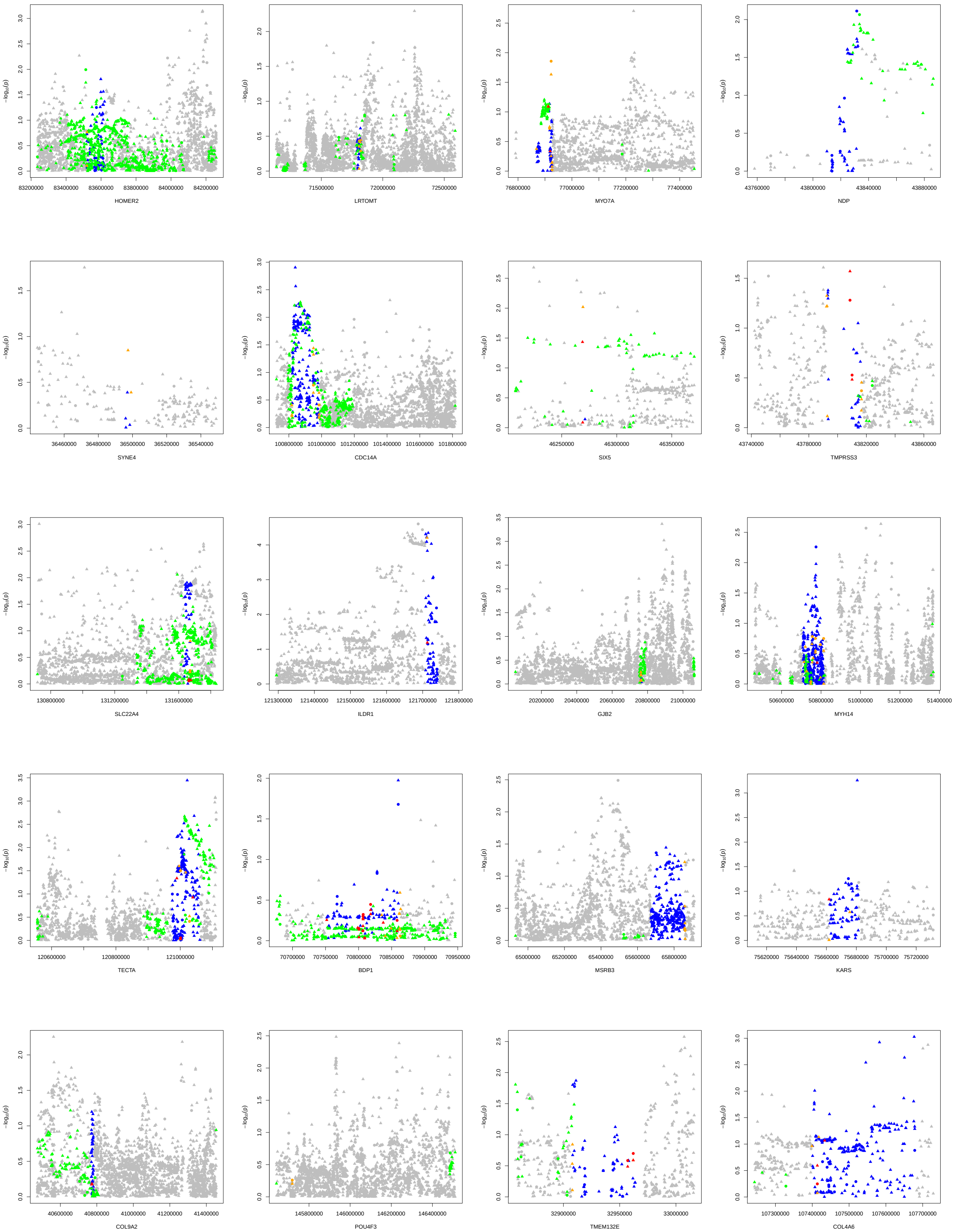
HOMER2
LRTOMT
MYO7A
NDP
SYNE4
CDC14A
SIX5
TMPRSS3
SLC22A4
ILDR1
GJB2
MYH14
TECTA
BDP1
MSRB3
KARS
COL9A2
POU4F3
TMEM132E
COL4A6

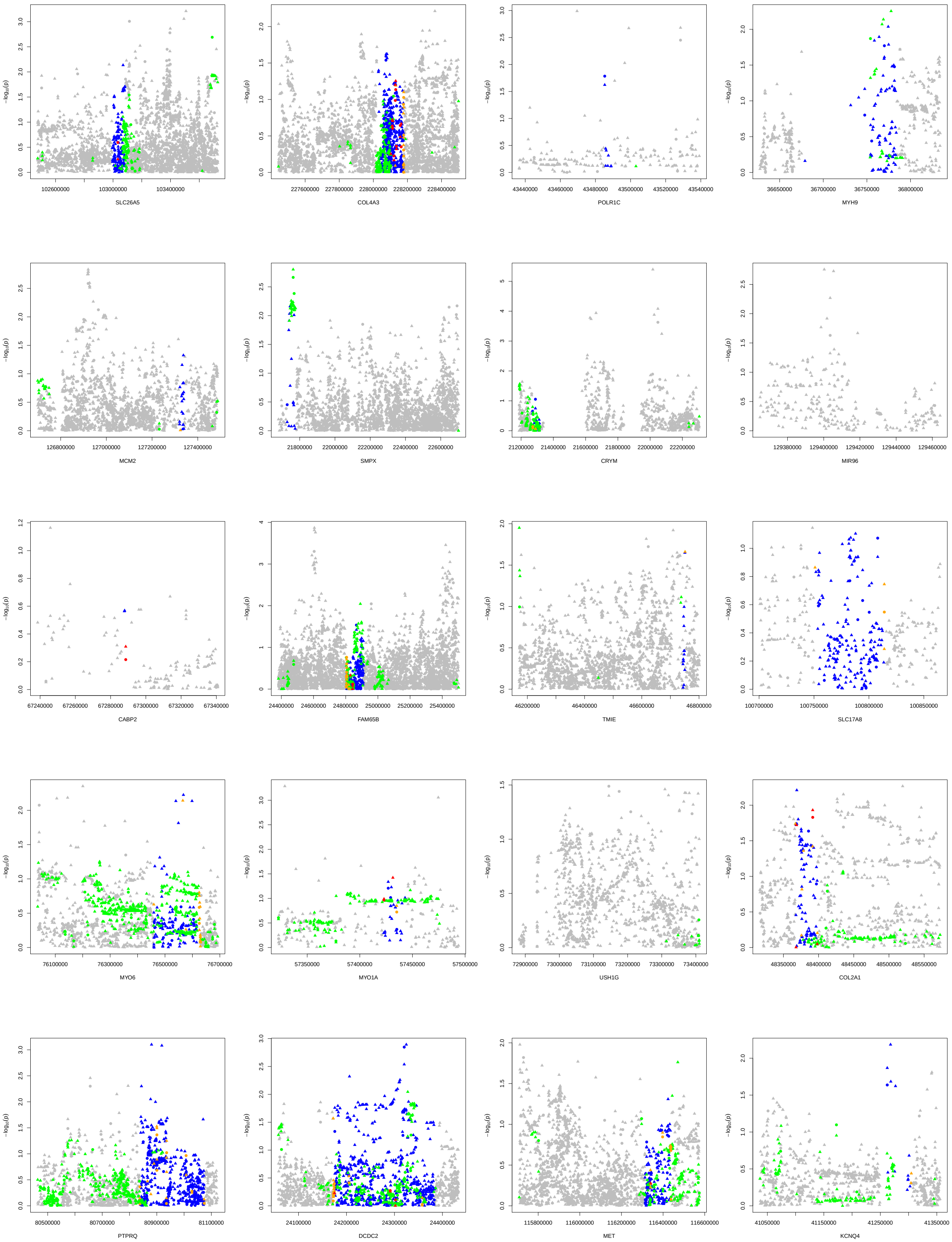

SLC26A5
COL4A3
POLR1C
MYH9
MCM2
SMPX
CRYM
MIR96
CABP2
FAM65B
TMIE
SLC17A8
MYO6
MYO1A
USH1G
COL2A1
PTPRQ
DCDC2
MET
KCNQ4
$-\log_{10}(p)$

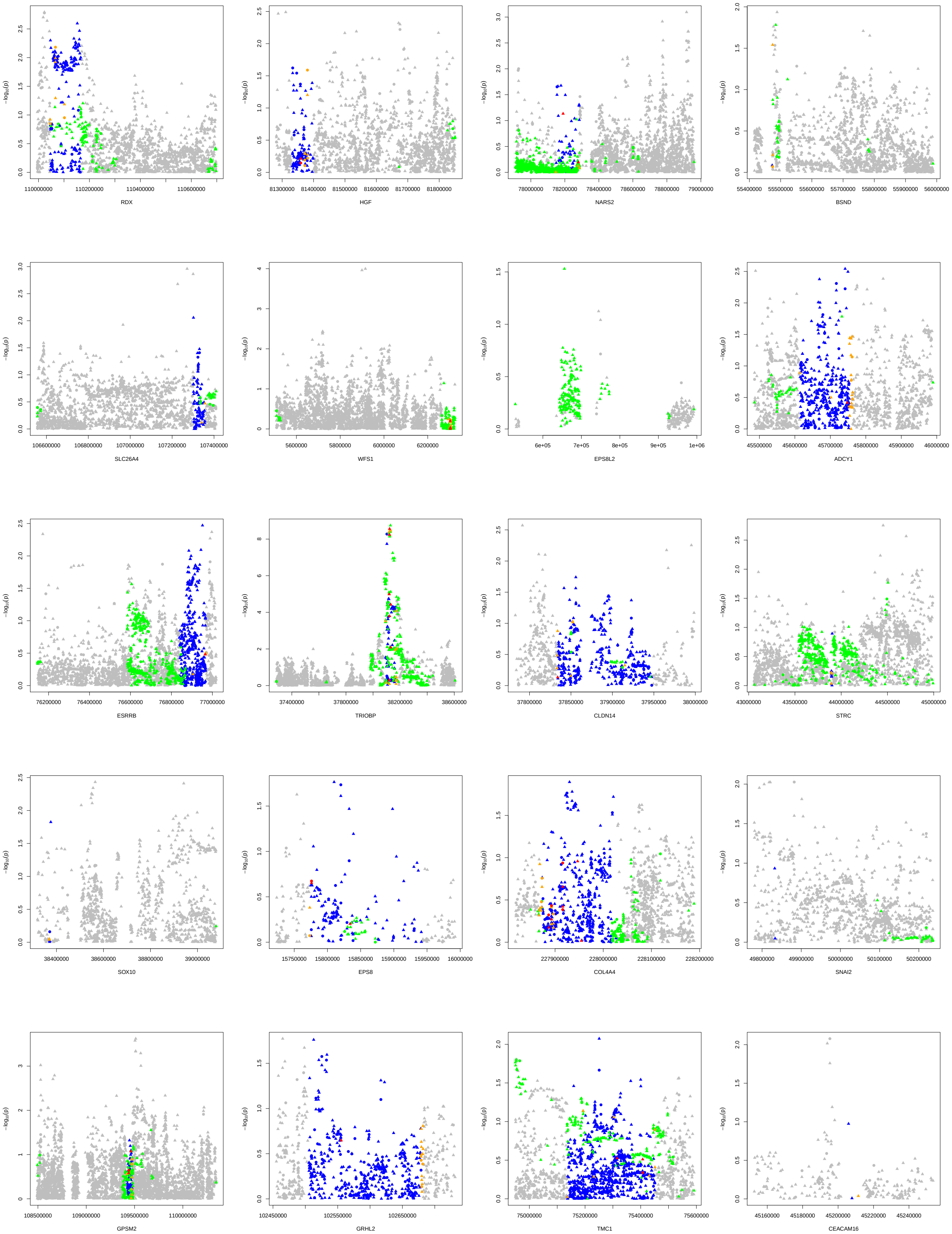
RDX
HGF
NARS2
BSND
SLC26A4
WFS1
EPS8L2
ADCY1
ESRRB
TRIOBP
CLDN14
STRC
SOX10
EPS8
COL4A4
SNAI2
GPSM2
GRHL2
TMC1
CEACAM16

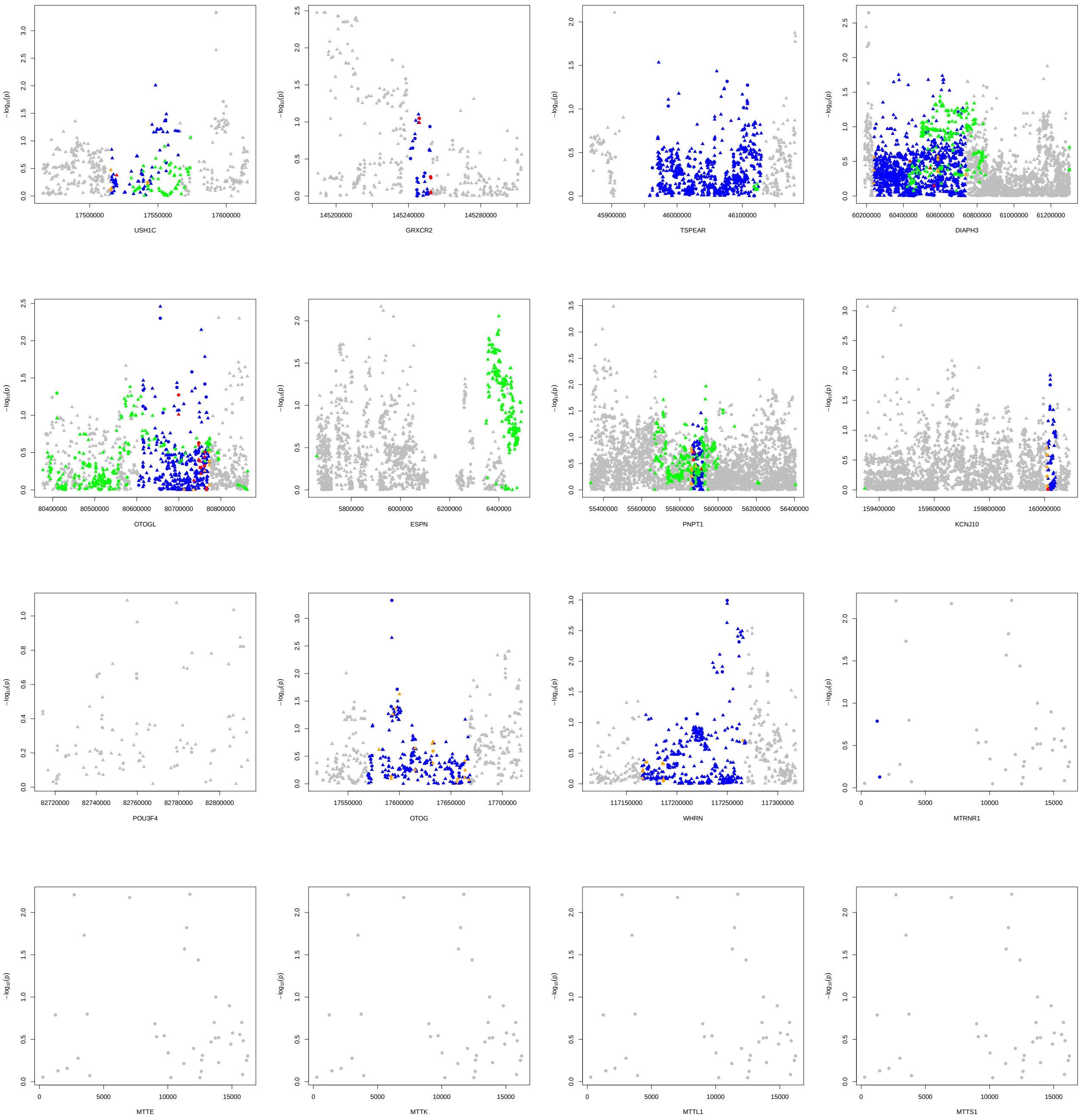
−log10(p)
USH1C
GRXCR2
TSPEAR
DIAPH3
OTOGL
ESPN
PNPT1
KCNJ10
POU3F4
OTOG
WHRN
MTRNR1
MTTE
MTTK
MTTL1
MTTS1
