# Supplementary material for: A Large Genome-Wide Association Study of Age-Related Hearing Impairment Using Electronic Health Records
Source: PLoS Genet. 2016 Oct 20;12(10):e1006371. doi: 10.1371/journal.pgen.1006371 (PMC5072625; doi:10.1371/journal.pgen.1006371)

S3 Fig. Age of onset distributions in GERA non-Hispanic white cases. Age of onset distributions for the ARHI variants in GERA non-Hispanic whites, based on residuals and normalized to a female without diabetes, hypertension, or osteoporosis.

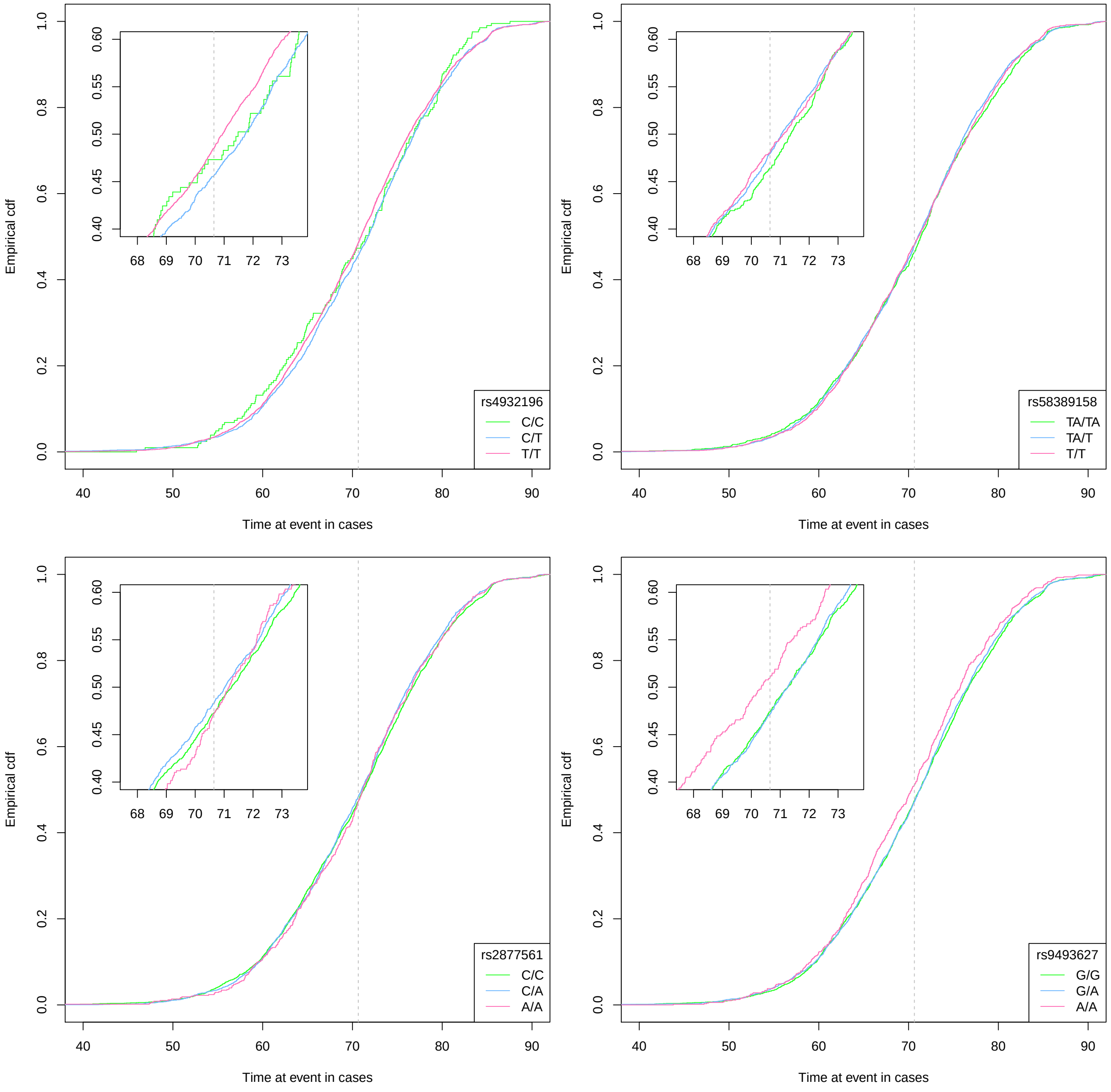

Supplement: S3 Fig — Age of onset distributions for the ARHI variants in GERA non-Hispanic whites, based on residuals and normalized to a female without diabetes, hypertension, or osteoporosis. (PDF) [file pgen.1006371.s004.pdf]
