# Supplementary material for: A Large Genome-Wide Association Study of Age-Related Hearing Impairment Using Electronic Health Records
Source: PLoS Genet. 2016 Oct 20;12(10):e1006371. doi: 10.1371/journal.pgen.1006371 (PMC5072625; doi:10.1371/journal.pgen.1006371)

S4 Fig. Mouse tissue expression of *ACAN* and *ISG20*. FACS, flourescence-activated cell sorting hair cells. E represents embryonic tissue, P postnatal. Numbers represent number of days.

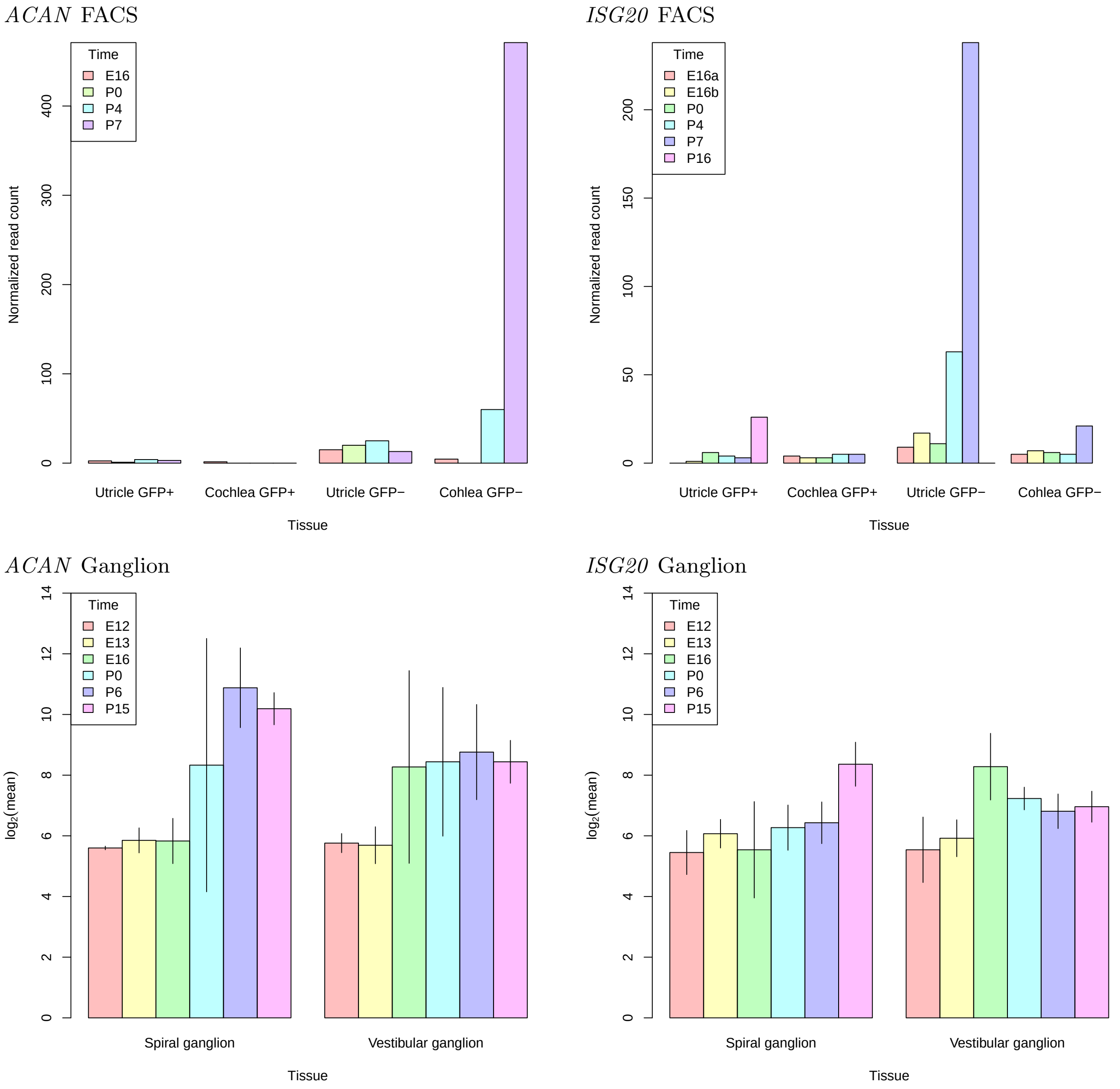

Supplement: S4 Fig — FACS, fluorescence-activated cell sorting hair cells. E represents embryonic tissue, P postnatal. Numbers represent numbers of days. (PDF) [file pgen.1006371.s005.pdf]
